# The Catalytic and Non-catalytic Functions of the *Brahma* Chromatin-Remodeling Protein Collaborate to Fine-Tune Circadian Transcription in *Drosophila*

**DOI:** 10.1371/journal.pgen.1005307

**Published:** 2015-07-01

**Authors:** Rosanna S. Kwok, Ying H. Li, Anna J. Lei, Isaac Edery, Joanna C. Chiu

**Affiliations:** 1 Department of Entomology and Nematology, College of Agricultural and Environmental Sciences, University of California, Davis, Davis, California, United States of America; 2 Center for Advanced Biotechnology and Medicine, Rutgers, the State University of New Jersey, Piscataway, New Jersey, United States of America; University of Massachusetts Medical School, UNITED STATES

## Abstract

Daily rhythms in gene expression play a critical role in the progression of circadian clocks, and are under regulation by transcription factor binding, histone modifications, RNA polymerase II (RNAPII) recruitment and elongation, and post-transcriptional mechanisms. Although previous studies have shown that clock-controlled genes exhibit rhythmic chromatin modifications, less is known about the functions performed by chromatin remodelers in animal clockwork. Here we have identified the *Brahma* (*Brm*) complex as a regulator of the *Drosophila* clock. In *Drosophila*, CLOCK (CLK) is the master transcriptional activator driving cyclical gene expression by participating in an auto-inhibitory feedback loop that involves stimulating the expression of the main negative regulators, *period* (*per*) and *timeless* (*tim*). BRM functions catalytically to increase nucleosome density at the promoters of *per* and *tim*, creating an overall restrictive chromatin landscape to limit transcriptional output during the active phase of cycling gene expression. In addition, the non-catalytic function of BRM regulates the level and binding of CLK to target promoters and maintains transient RNAPII stalling at the *per* promoter, likely by recruiting repressive and pausing factors. By disentangling its catalytic versus non-catalytic functions at the promoters of CLK target genes, we uncovered a multi-leveled mechanism in which BRM fine-tunes circadian transcription.

## Author Summary

The circadian clock is an endogenous timing system that enables organisms to anticipate daily changes in their external environment and temporally coordinate key biological functions that are important to their survival. Central to *Drosophila* clockwork is a key transcription factor CLOCK (CLK). CLK activates expression of target genes only during specific parts of the day, thereby orchestrating rhythmic expression of hundreds of clock-controlled genes, which consequently manifest into daily rhythms in physiology and behavior. In this study, we demonstrated that the *Brahma (Brm)* chromatin-remodeling protein interacts with CLK and fine-tune the levels of CLK-dependent transcription to maintain the robustness of the circadian clock. Specifically, we uncovered two distinct but collaborative functions of *Brm*. *Brm* possesses a non-catalytic function that negatively regulates the binding of CLK to target genes and limits transcriptional output, likely by recruiting repressive protein complexes. Catalytically, *Brm* functions by condensing the chromatin at CLK target genes, specifically when transcription is active. This serves to precisely control the level of repressive factors likely recruited by *Brm* as well as other transcriptional regulators. By disentangling these two roles of *Brm*, our study uncovered a multi-layered mechanism in which a chromatin remodeler regulates the circadian clock.

## Introduction

Circadian clocks are endogenous timekeeping mechanisms that drive rhythms in physiology and behavior with an approximately 24-hour period, allowing organisms from all kingdoms of life to anticipate and operate through predictable daily environmental changes. Much progress has been made in understanding the architecture of the molecular oscillators regulating these cell-autonomous clocks in different organisms, and the mechanisms in which the core oscillators communicate temporal information via circadian transcription that ultimately drives many overt physiological rhythms [1–3]. In *Drosophila melanogaster*, two basic helix-loop-helix PER-ARNT-SIM (bHLH-PAS) transcription factors CLOCK (CLK) and CYCLE (CYC) are at the core of the molecular oscillator, which operates through two interlocked transcriptional-translational feedback loops [1]. In the major loop, CLK and CYC form heterodimers and bind to E-box regulatory elements on genes encoding the main negative factors PERIOD (PER) and TIMELESS (TIM) that inhibit the transcriptional activity of CLK-CYC, and consequently their own transcription, closing off one autoregulatory feedback circuit. CLK-CYC also initiate a second loop by activating the transcription of genes encoding regulators of *Clk* expression [4–5]. VRILLE (VRI), a basic leucine zipper (bZIP) transcription factor, binds to D-box (also called V/P box) elements on the *Clk* promoter to repress *Clk* activation by PAR Domain Protein 1ε (PDP1ε).

The temporal control in the expression levels of these key oscillating mRNAs and proteins over the circadian cycle is critical for the normal progression of the clock. CLK activation of *per*, *tim*, *vri*, and *pdp1ε* occurs in late day and peaks in the early evening. Post-transcriptional and post-translational regulatory mechanisms create a time-delay, causing the levels of these proteins to peak about 6 hours later in mid to late evening [6–13]. As PER and TIM proteins accumulate and enter the nucleus, they dimerize and repress the activity of CLK-CYC. This repression is relieved upon sunrise due to the degradation of light-sensitive TIM [14] and subsequently PER a few hours later through the proteasome pathway [15–16], thus initiating another round of CLK-CYC-mediated transcription. On the other hand, the expression of *Clk* is antiphase, first initiating during the late evening and peaking in the early morning. The precise timing of *Clk* expression is the result of differential protein accumulation of VRI and PDP1ε due to yet undiscovered mechanisms [4–5]. Whereas there is little delay in VRI accumulation following *vri* mRNA production, there is a 3 to 6 hour delay in PDP1ε accumulation, postponing prominent accumulation of *Clk* mRNA until early to mid-day. Two other clock components, *clockwork orange (cwo*) [17–18] and *nejire (nej)*/CREB-binding protein (CBP) [19–20], have also been shown to regulate CLK-dependent transcription, but the exact mechanisms are still controversial. Outside of the core oscillator, CLK-CYC have been found to bind more than 800 downstream target genes, leading to their rhythmic transcription [21].

Since the observations of chromatin modifications occurring as a result of light resetting [22] and rhythmic histone acetylation in circadian promoters [23] in mammalian clock systems, the importance of chromatin remodeling in modulating the activity of clock transcription factors and circadian transcription in animal clocks, as well as clocks in other organisms, is becoming increasingly evident [24–25]. Gene-specific and genome-wide studies have now established that many clock-controlled genes (ccgs) exhibit rhythmic chromatin modifications. For example, many hallmarks of transcriptional activation such as H3K9ac and H3K4me3 have been found to coincide with rhythmic CLK-CYC binding to the E-boxes of *per* and *tim* in *Drosophila* [26] as well as CLOCK-BMAL1 dependent transcription in mammals [27–28]. Many of the histone modifying proteins and their roles in regulating circadian transcription have now been explored (reviewed in [24–25]). In addition to histone modifiers, ATP-dependent chromatin remodelers can regulate transcription factor accessibility to DNA through mechanisms such as nucleosome reorganization [29–31]. This aspect of chromatin remodeling in clock systems has been studied most extensively in *Neurospora crassa*. Key transcription factors WHITE COLLAR-1 (WC-1) and WHITE COLLAR-2 (WC-2) were observed to bind differentially to the *frequency (frq)* promoter as chromatin structure is altered, and a number of ATP-dependent chromatin remodelers including CLOCKSWITCH (CSW), chromodomain helicase DNA-binding (CHD-1), and Clock ATPase (CATP), have been identified to play a role in this process [32–34]. Whereas CSW and CHD-1 have been shown to facilitate the downregulation of *frq* transcription, CATP is believed to decrease nucleosome density, increase WCC (WHITE COLLAR complex) binding to *frq*, and promote *frq* activation. Recently, an additional ATP-dependent remodeler, SWI/SNF (SWItch/Sucrose NonFermentable), has been implicated in remodeling chromatin to allow for activation of *frq* [35].

Despite the growing realization that nucleosome reorganization is an important aspect of regulating circadian transcription, how it contributes to animal clocks is not well understood. *Kismet (kis*), a subunit of an ATP-dependent remodeler has been shown to be involved in light entrainment in the *Drosophila* circadian timing system, but does not seem to affect the core oscillator [36]. CLOCK has recently been found to act as a pioneer transcription factor to open up the chromatin in mouse liver clocks to facilitate binding of additional transcriptional factors on CLOCK target genes, but the mechanism still needs to be characterized [28]. To better understand the regulatory role of chromatin remodeling in the *Drosophila* clock, we used a proteomic approach and screened for CLK interactors that are involved in nucleosome organization. Among the partners we discovered was the *Brahma* (*Brm*) (SWI/SNF class) chromatin-remodeling protein complex. Similar to the SWI/SNF complex originally identified in yeast, the orthologous complex in animals also possesses chromatin remodeling activity and is implicated in a variety of cellular processes including differentiation, proliferation, and DNA repair [29]. In *Drosophila*, the *Brahma* complex is comprised of multiple protein components, in which the *brm* gene encodes BRM, the catalytic subunit containing the ATPase responsible for hydrolysis of ATP to mobilize nucleosomes [37–38]. In addition to an ATPase domain, BRM has a bromodomain [37], which can recognize and bind to acetylated lysine residues on histone tails [39–40], hence its close association with transcriptional activation. Nonetheless, BRM activity has also been associated with repression depending on target genes and cell types [41]. Here we characterize the role of BRM in regulating the *Drosophila* circadian clock through modifications of the chromatin landscape. We also differentiate between its catalytic function in modifying chromatin structure and its non-catalytic function, leading to downstream effects on transcription factor binding, RNA polymerase II (RNAPII) occupancy, clock gene expression, and overt behavioral rhythms. Our studies suggest that the SWI/SNF (*Brahma*) complex has a key regulatory role in eukaryotic clocks and provide novel insights into how animal clocks are regulated by chromatin remodeling.

## Results

### The *Brahma* complex interacts with key clock transcription factors and regulates the *Drosophila* circadian clock

**Fig 1:**
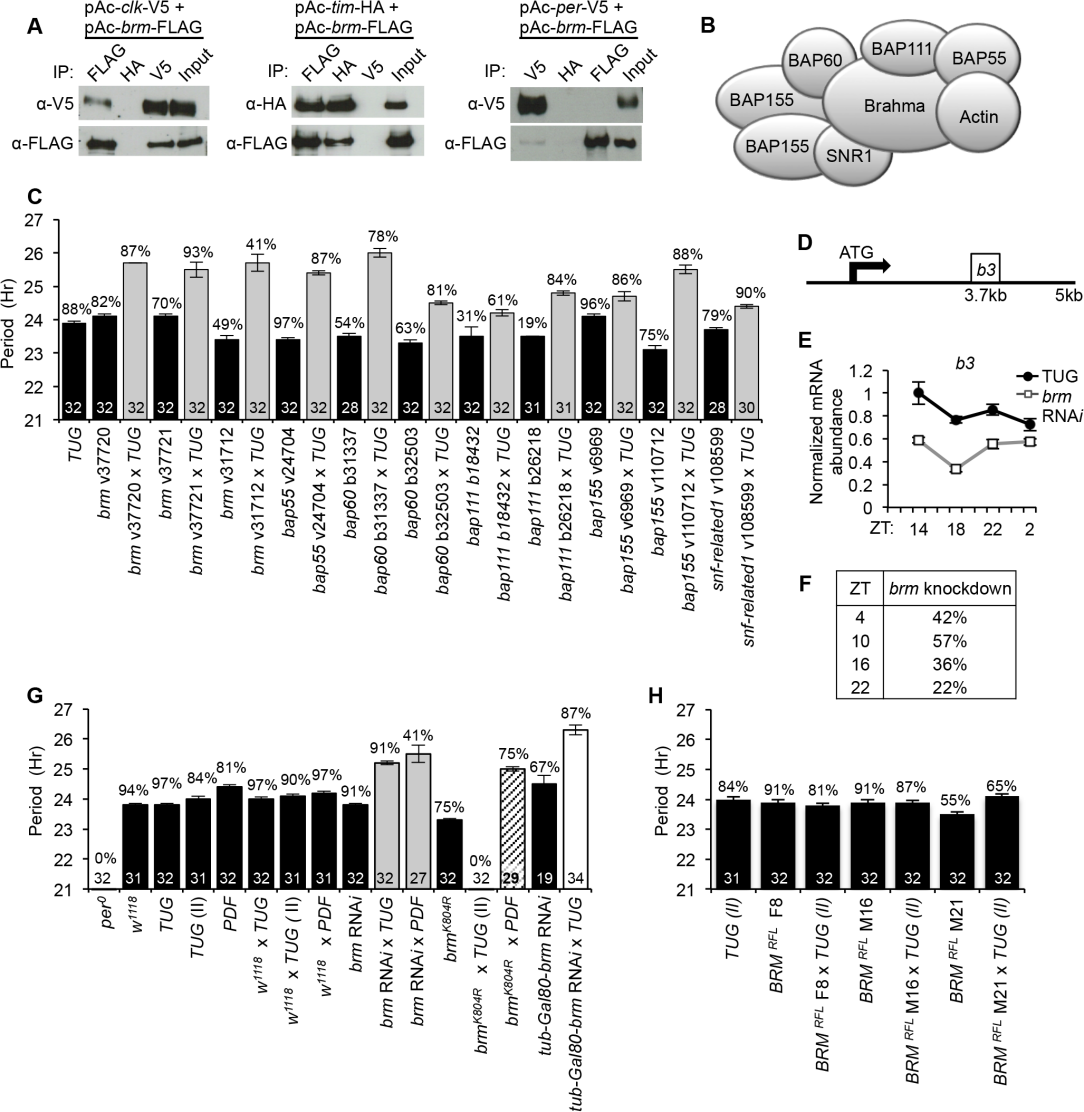
The *Brahma* protein complex regulates circadian clock function in *Drosophila*. *(A)* Western blots showing results of reciprocal co-IP assays to detect interaction of BRM with CLK, PER, and TIM in *Drosophila* S2 cells. Proteins extracted from S2 cells were either immunoprecipitated with α-FLAG to pull down BRM, α-HA to pull down TIM, and α-V5 to pull down CLK or PER. A negative control co-IP was performed for each pair of interactions using an antibody recognizing an epitope tag that is not present in the two proteins of interest. Immuno-complexes were subjected to western blotting to detect the bait protein or protein interactions. Input for the co-IP is indicated. All experiments were repeated at least three times. *(B)* Schematic of the core *Brahma* (SWI/SNF) chromatin-remodeling complex showing multiple subunits [29]. All subunits, except Actin, were targeted for RNA*i* knockdown in behavioral assays. *(C)* Knockdown of individual *Brahma* complex subunits in *tim*-expressing neurons results in period lengthening. Shown are circadian periods for free running behavioral rhythms in constant dark conditions for males of the indicated genotypes. Analysis of period length was performed using Cycle P in the FaasX software package. Black bars represent control parental driver lines. Grey bars represent RNA*i* knockdown in *tim*-expressing neurons using the *tim-UAS-Gal4* driver (*w; UAS-dicer 2; TUG*). When available, multiple responder lines were used for RNA*i* knockdown for individual genes. Stock numbers for responder lines are indicated. Stocks starting with “v” were from VDRC, while stocks starting with “b” were from Bloomington Stock Center. Numbers within bars indicate sample size. Percentages above bars indicate percentage of flies displaying rhythmicity. Error bars = SEM. *(D)* Schematic of *brm* primer (b3) placement for quantitative PCR to assay extent of *brm* knockdown. *(E)* Expression of *brm* in control *TUG* parental driver line and *brm* RNA*i* mutant (v37720 X *TUG*) as assayed over a circadian day using quantitative PCR. Error bars = SEM of technical triplicates. Results are representative of two biological replicates. *(F)* Percent of *brm* knockdown in RNA*i* shown in (E) relative to *brm* expression in *TUG* parental control at corresponding time points. *(G)* Results of behavioral assays to determine circadian periodicity with similar format as described in (C). *brm* RNA*i* (v37720) was expressed in PDF neurons (*w; UAS-dicer 2*; *pdf-gal4* X v37720) as well as in *tim*-expressing neurons (grey bars). A dominant-negative *brahma* (*brm* ^*K804R*^ *)* transgene was expressed in *tim*-expressing cells (*TUG(II)*) or PDF neurons (*pdf-gal4*) (striped bars). Inhibition of *brm* RNAi expression during development using temperature-sensitive GAL80 system still resulted in a period-lengthening phenotype (white bar). *(H)* Three independent transgenic *BRM* ^*RFL*^ transformants (BRM overexpression) display normal circadian phenotypes when crossed with the *TUG(II)* driver. Error bars = SEM.

To identify chromatin-remodeling proteins that regulate CLK-dependent transcription in *Drosophila*, we performed a label-free quantitative proteomic screen using affinity purification followed by tandem mass spectrometry (MS/MS) to identify CLK-interacting proteins in *Drosophila* Schneider 2 (S2) tissue culture cells (Lam et al. in preparation). We focused on the interaction between BRM and CLK, rather than BRM and CYC, as CLK has been shown to be the limiting factor in CLK-CYC dependent transcriptional activation [42] and ectopic clocks can be generated by misexpressing CLK alone, indicating its central role in circadian gene activation [43]. Prior work has shown that exogenous expression of CLK in S2 cells can interact with endogenous CYC to activate circadian promoters and is also responsive to the inhibitory effects of PER-TIM, indicating that the core CLK-dependent transcriptional machinery can operate in S2 cells [44]. Cytoplasmic and nuclear extracts were prepared from S2 cells expressing recombinant versions of CLK modified with epitope tags to facilitate purification. Our quantitative proteomic pipeline identified multiple subunits of the *Brahma* complex as significant interactors of CLK, specifically when probing nuclear extracts. We validated our pull-down of CLK with endogenous BRM, the catalytic subunit of the complex, by co-expressing epitope-tagged versions of both proteins in S2 cells followed by immunoprecipitation (IP). Western blot analysis of reciprocal co-IPs indicated strong interaction of BRM to CLK (Fig 1A). In addition, interactions of BRM to negative factors PER and TIM were also tested. Whereas TIM was found to interact with BRM to a similar extent as interactions between BRM and CLK, the interaction between BRM and PER was detectable, but much weaker (Fig 1A).

Co-IP experiments using proteins extracted from whole fly heads were also performed to confirm the interactions observed in S2 cells (S1 Fig). To facilitate analyzing BRM in flies, we generated responder transgenic lines that express BRM carrying a 3X FLAG epitope tag (*UAS-BRM* ^*RFL*^) and directed their expression using a *tim(UAS)-gal4* driver targeting *tim*-expressing cells, herein termed *TUG(II)* [45]. These flies will be discussed in more details below. Whereas BRM was found to interact with CLK when CLK target gene transcription is active (ZT8 to ZT20) (S1A Fig middle panel and S1C Fig), its interaction with TIM appeared stronger at ZT20, during the downswing of the transcription cycle (S1A Fig bottom panel and S1D Fig). In addition, BRM appears to interact preferentially with hypophosphorylated CLK (S1E Fig), which is the predominant isoform bound to target gene regions during active transcription [46–48]. Interaction of BRM and PER was not observed in fly heads, consistent with results obtained in S2 cells (Fig 1A).

We next determined if the *Brahma* complex is involved in regulating the circadian oscillator and generating ~24 hour rhythms. We used RNA*i* to individually knock down the expression of each of the core subunits (Fig 1B) in *tim*-expressing clock neurons (*TUG*) and performed fly locomotor activity assays to detect abnormal free-running rhythms. Following entrainment under standard conditions of 12hr light:12hr dark (LD) at 25°C, flies in which individual *Brahma* complex subunits (*Brahma*, *Bap55*, *Bap60*, *Bap111*, *Bap155* or *Moira*, *and Snf-related 1*) were knocked down by RNA*i* showed period-lengthening to a similar extent (1 to 2 hours longer relative to parental control) when placed into constant dark (DD) conditions (Fig 1C). This suggests that the *Brahma* complex is necessary for sustaining a normal circadian period, and that knockdown of one subunit may be sufficient in impairing the function of the entire complex within the context of clock function. Quantitative PCR (qPCR) analysis using RNA extracted from whole fly heads revealed a roughly 40% knockdown of *brm* in the flies expressing *brm* RNA*i* in *tim*-expressing cells (Fig 1D–1F). However, since *brm* is involved in many physiological processes and is expressed in many cell types in addition to *tim*-expressing cells, we expect that the actual level of *brm* knockdown to be greater within *tim*-expressing cells. To support a role for the *brm* complex in circadian regulation, we also used *pdf-gal4* to drive *brm* RNA*i* expression in a smaller subset of clock neurons that are important for establishing circadian period and observed a similar period-lengthening effect (Fig 1G).

As *brm* has been shown to be involved in development [37, 49], we sought to rule out developmental defects from decreased *brm* expression contributing to the observed behavioral phenotypes. To this end, we utilized a temperature-sensitive GAL80 system (*UAS-tub-Gal80* ^*ts*^) to repress activation of the responder *brm-RNAi* transgene by GAL4 during development by maintaining flies at 18°C until 3 days before the locomotor activity assay. We then transferred the flies to 29°C to relieve the GAL80 repression and initiate *brm* RNA*i* expression. The behavioral assay was performed at 29°C and *brm* RNA*i* knockdown in clock neurons still resulted in period-lengthening (Fig 1G), thus ruling out contributions from developmental effects. Finally, as an independent method to knockdown BRM function, we assayed flies expressing *brm* ^*K804R*^ in *tim*-expressing clock cells (*UAS*-*brm* ^*K804R*^ X *TUG(II)*). The *brm* ^*K804R*^ transgene encodes a catalytic inactive mutant of BRM that contains a lysine to arginine substitution in the ATP-binding site [50]. Flies expressing *brm* ^*K804R*^ exhibited more severe circadian rhythm abnormalities as compared to *brm* RNA*i* knockdown when both transgenes were driven with the *TUG* driver (Fig 1G). Closer inspection of their behavioral rhythms revealed that *brm* ^*K804R*^ mutants displayed period-lengthening in the first two days into DD as in the case of the *brm* RNA*i* knockdown, which quickly deteriorated into arrhythmicity starting DD3 (S2 Fig).

### BRM localizes to CLK binding sites on *per* and *tim*

Since we observed BRM interacting with CLK and showed that *brm* knockdown and mutation affect oscillator function, we hypothesized that BRM may be interacting with CLK at its target genes to regulate expression. To examine if BRM binds to CLK targets, we sought to assay BRM binding to E-box elements at the promoters of *per* and *tim*. We generated the *UAS-BRM* ^*RFL*^ responder and directed its expression using *TUG(II)*. Introduction of the FLAG-*brm* transgene did not alter circadian clock function as assayed by behavioral rhythms using three independent responder lines (Fig 1H). This is perhaps not surprising since BRM protein is normally incorporated into a multi-subunit protein complex (Fig 1B), and overexpression of *brm* alone might not lead to increase in functional *Brm* complexes. This suggests that these transgenic flies can be used as tools to examine BRM binding to CLK target genes. In addition, the FLAG epitope tag has been used successfully for ChIP analysis in studying *Drosophila* clocks [21].

**Fig 2:**
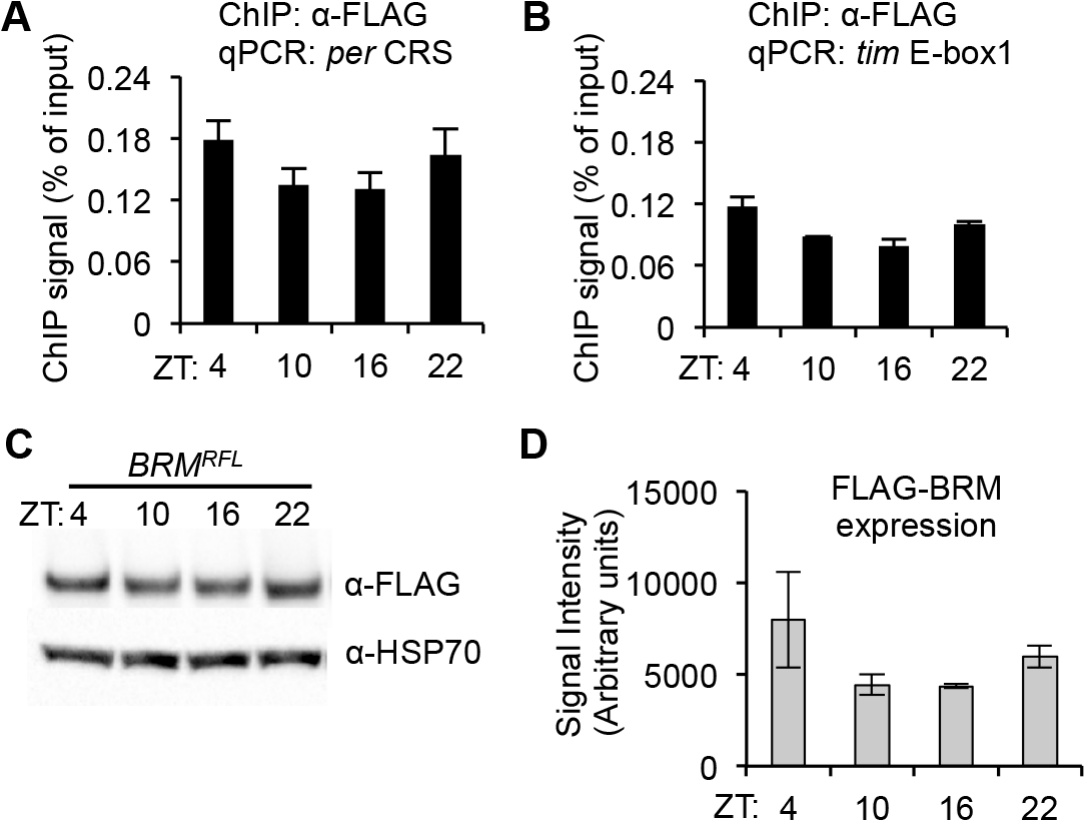
BRM localizes on CLK binding sites at the *per* and *tim* genes. ChIP assay showing BRM localization to *(A) per* CRS and *(B) tim* E-box 1. BRM is tagged with FLAG epitope (*BRM* ^*RFL*^) and expressed using the UAS/GAL4 system in *tim*-expressing neurons (M16 line is used for all ChIP experiments). Flies were harvested at the four indicated time points (ZT). α-FLAG was used to pull down FLAG-tagged BRM. Data shown are from three biological ChIP replicates, with technical triplicates performed during qPCR for each biological replicate. Non-specific BRM binding was detected by amplifying an intergenic region (FBgn0003638) of the *Drosophila* genome and subtracted from the signal from the *per* CRS and *tim* E-box 1 signals. Error bars = SEM of biological replicates. *(C)* Western blot showing FLAG-BRM expression in *BRM* ^*RFL*^ flies at four time points (ZT) over a circadian cycle as detected using α-FLAG suggests that the amount of BRM binding reflects protein levels of FLAG-BRM. *(D)* Quantification of FLAG-BRM expression using HSP70 for normalization. Data plotted are from quantifications of two separate biological replicates. Error bars = SEM.

ChIP followed by qPCR analysis of flies expressing *BRM* ^*RFL*^ revealed that over the circadian cycle, BRM is constitutively bound to the fifth E-box element at the *per* promoter (Fig 2A), which has previously been identified as a CLK binding site for transcriptional activation [26, 51] and is within a 69 bp clock regulatory sequence (CRS), a region critical in generating the oscillatory patterns of *per* expression [52–53]. Additionally, BRM was found to bind to the first E-box element on the *tim* locus (Fig 2B), which has also been identified as a CLK binding site [26]. Control ChIP experiments were also performed on flies that do not express FLAG-tagged transgene to rule out the possibility of non-specific binding (S3 Fig). It is worth noting that BRM localization on circadian E-box elements appears weakly rhythmic. However, upon quantification of FLAG-BRM expression (Fig 2C and 2D), it seems that its protein levels at ZT4 and ZT22 are slightly elevated relative to levels found at ZT10 and ZT16, suggesting that the level of BRM binding to *per* and *tim* E-box elements may reflect expression levels of FLAG-BRM. Quantitative PCR analysis of endogenous *brm* expression did not reveal any cycling of *brm* steady state mRNA (refer to Fig 1E), suggesting the weak rhythmicity in FLAG-BRM binding to *per* and *tim* promoters may be the result of driving FLAG-BRM expression using a *tim* driver. For the target genes we tested (*per* and *tim)*, we observed preferential binding to the promoters over gene bodies (S4 Fig), consistent with binding patterns of BRM in both *Drosophila* and mammals genes [54–55].

### BRM plays an inhibitory role in clock gene transcription by increasing nucleosome density at *per* and *tim* promoters

**Fig 3:**
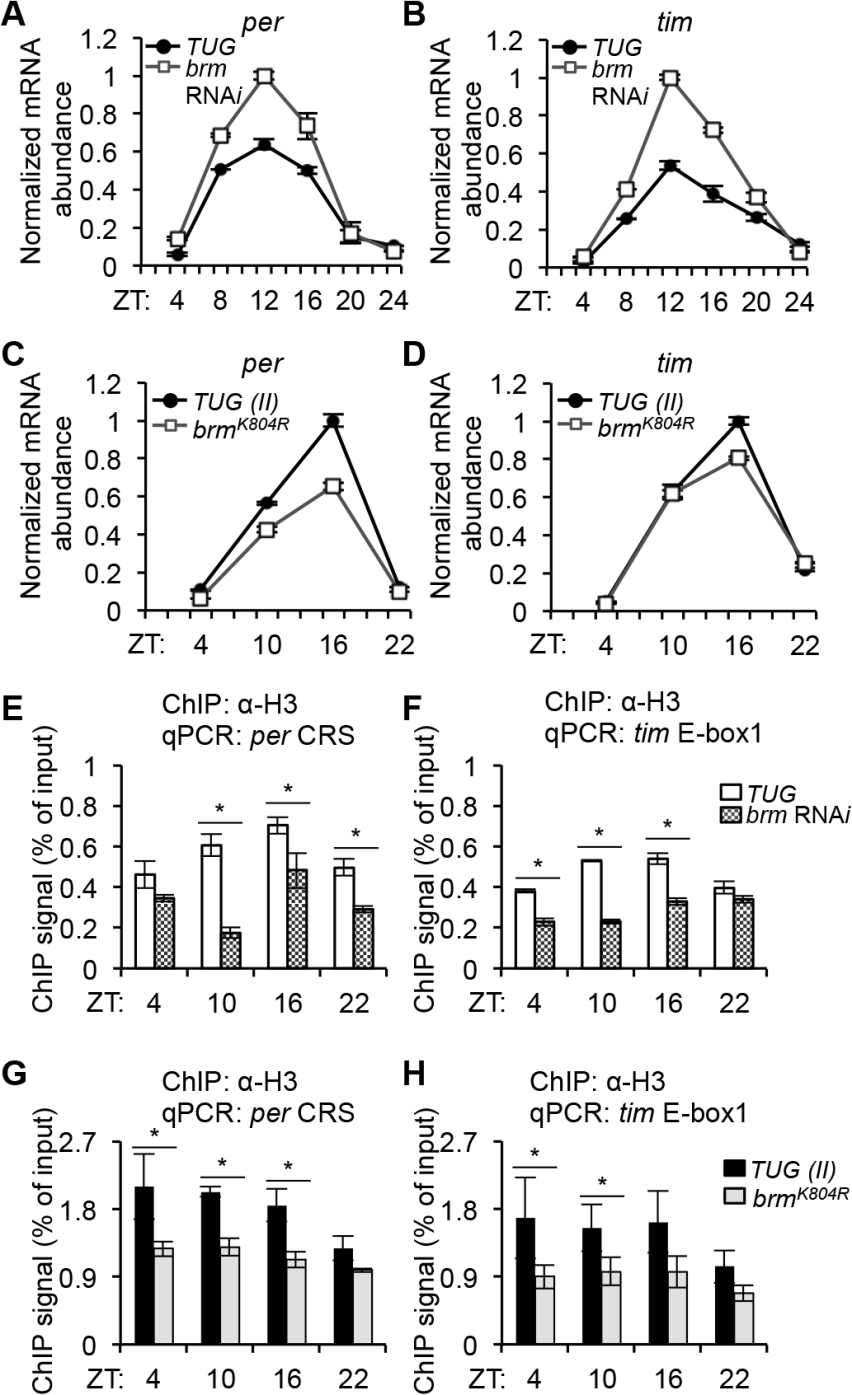
*Brahma* regulates nucleosome occupancy and transcriptional output at *per* and *tim* promoters. Expression of *(A) per* and *(B) tim* was assayed in flies expressing *brm* RNA*i* in *tim*-expressing neurons (*brm* RNA*i* v37720 X *TUG*) compared to control parental line (*TUG*). Expression of *(C) per* and *(D) tim* was assayed in flies expressing the *brm* ^*K804R*^ transgene in *tim*-expressing cells (*UAS*-*brm* ^*K804R*^ X *TUG(II)*) and compared to control *TUG(II*) lines. All gene expression analysis was performed with quantitative real-time PCR using SYBR green chemistry. Steady state mRNA levels at six time points (ZT) for *(A)* and *(B)* and four time points for *(C)* and *(D)* over a circadian cycle were normalized to non-cycling *cbp20* levels, and expressed as a fraction of the peak expression level (peak = 1). Experiments were performed at least 3 times and representative results are shown. Error bars = SEM for technical triplicates for each biological replicate. *(E-H)* Corresponding histone H3 ChIP assays for the same genotypes of flies used for gene expression analysis as described in *(A-D)* illustrating decrease of nucleosome occupancy at the *per (E* and G) and *tim* (*F* and *H*) promoters in the *brm* mutants (*brm* RNA*i* and *brm* ^*K804R*^). Error bars = SEM (n = 3) for biological replicates. Asterisks denote significant difference between control and *brm* knockdown at indicated time points (P < 0.05).

The BRM complex has previously been shown to have either stimulating or repressive effects on gene expression depending on target genes by altering DNA-histone contacts in *Drosophila* developmental pathways [41, 56, 57]. Since we found that BRM interacts with hypophosphorylated CLK, our initial hypothesis was naturally that BRM cooperates with CLK to facilitate activation of CLK targets such as *per* and *tim*, perhaps by opening up the chromatin. This is an attractive hypothesis for two reasons. First, CLK has been labeled a pioneer transcription factor with the ability to bind DNA-wrapped nucleosomes and subsequently recruit additional factors to de-condense the chromatin to perpetuate transcriptional activation [28]. Moreover, the SWI/SNF complex in *Neurospora* has recently been shown to decrease nucleosome density, helping to activate *frq* transcription [35]. Using qPCR, we assayed steady state mRNA levels of various CLK-dependent transcripts (*per*, *tim*, *vri*, *pdp1ε*) extracted from heads of flies expressing *brm* RNA*i* as compared to control flies (Figs 3A and 3B and S5A). Surprisingly, we found that there was an increase in the levels of all transcripts tested in flies expressing *brm* RNA*i*, especially during time points when these genes are normally actively transcribing (ZT 8 to 16). We also analyzed nascent pre-mRNA levels, focusing specifically on *per* and *tim*, and observed a similar extent of increase (S6A Fig). This initial evidence suggests that BRM may have a repressive role in limiting CLK-dependent transcription in the *Drosophila* clock. Since CLK-dependent transcription is still being effectively repressed starting in the early evening in flies in which *brm* is knocked down by RNA*i*, the role of BRM may be limited to preventing excessive transcription during the active phase, and not to initiate and maintain transcriptional repression. In support of our hypothesis that BRM has a repressive role, it is noteworthy that in a recent genome-wide study, inactivation of two core subunits (SNF5 and BRG1) of the *Brahma* complex in mammalian cells resulted in variable gene expression outcomes, with more genes being upregulated rather than downregulated [55].

In the same study [55], inactivation of *Brahma* subunits and upregulation of a large subset of genes correlated with reduced nucleosome occupancy, especially in the peri-TSS (transcription start site) region. We therefore investigated whether the increased gene expression observed in circadian transcripts (Figs 3A and 3B, S5A and S6A) in flies expressing *brm* RNA*i* can be attributed to a decrease in nucleosome occupancy by measuring histone H3 density using ChIP-qPCR on *per* and *tim* promoters (E-boxes). At all time points tested, we observed a decrease in H3 density in the *brm* RNA*i* flies as compared to the control (Fig 3E and 3F). We performed the same experiment using flies expressing the catalytically-inactive BRM^K804R^ protein in *tim*-expressing cells and observed the same decrease in H3 density at the *per* and *tim* promoters (Fig 3G and 3H), indicating that the normal catalytic function of BRM at these promoters may be to increase nucleosome density. Interestingly, in the *TUG* control flies, histone H3 density at both the *per* and *tim* promoters is higher at ZT10 and 16, when transcription at these promoters are active, and is lower in the mid to late night when transcription is strongly repressed (ZT22). Although not as prominent, H3 density was also higher at ZT10 and 16 compared to ZT22 in the *TUG(II)* control flies for the data set to examine H3 density in flies expressing *brm* ^*K804R*^ (Fig 3G and 3H). The variance stemming from three biological replicates for the control *TUG(II)* flies at ZT4 were greater than at other time points tested (Fig 3G and 3H), and might have masked the weak rhythmicity in H3 density that appeared more prominent in the *TUG* control flies (Fig 3E and 3F). In general, the effects of *brm* RNA*i* and *brm* ^*K804R*^ on H3 density were not as prominent at ZT22, further suggesting that BRM does not play a major role during the strong repression phase of clock gene expression. Taken together, the results suggest that BRM may function to fine-tune clock gene expression by contributing to rhythmic changes in the chromatin landscape, leading to increased nucleosome occupancy during the active phase of CLK-mediated transcription.

Since flies expressing *brm* ^*K804R*^ showed decreased H3 density as compared to control flies, similar to the results for flies expressing *brm* RNA*i*, we anticipated that expression of *brm* ^*K804R*^ would also lead to an increase in mRNA expression of CLK target genes. Curiously, when we assayed clock gene expression using qPCR in flies expressing *brm* ^*K804R*^, we observed decreased clock gene expression with the time of peak expression remaining unchanged as compared to the control (Figs 3C and 3D and S5B). Analysis of nascent *per* and *tim* pre-mRNA levels using conventional qPCR did not show consistent decrease in expression, however analysis using droplet digital PCR (ddPCR), which has higher resolution, revealed small but significant decreases in nascent *per* and *tim* pre-mRNA expression in the *brm* ^*K804R*^ mutant (S6B Fig). The small decrease in *per* and *tim* transcripts observed in flies expressing *brm* ^*K804R*^ as compared to control when measuring pre-mRNA and the relatively larger decrease observed when measuring steady state mRNA suggests that the regulation of *per* and *tim* expression may be affected in the *brm* ^*K804R*^ mutant at both the transcriptional and posttranscriptional level.

The *brm* ^*K804R*^ allele encodes a catalytically inactive BRM protein [50], suggesting that this mutant may represent a valuable tool to disentangle the catalytic and non-catalytic functions of BRM in regulating clock gene expression. The most parsimonious explanation for why both *brm* RNAi and *brm* ^*K804R*^ lead to reductions in nucleosome occupancy is that they reduce the amount of endogenous catalytically active BRM that can bind chromatin and increase nucleosome density. In the case of the *brm* ^*K804R*^ mutant, the catalytically inactive BRM^K804R^ protein may be incorporated into endogenous *Brm* complexes to impair their catalytic function. However, the fact that *brm* ^*K804R*^ does not lead to an increase in clock gene expression suggests that once bound to chromatin, the catalytically inactive BRM^K804R^ protein functions in an inhibitory manner to repress transcription despite a more open chromatin landscape, perhaps by recruiting additional inhibitory factors. Thus, BRM likely modulates CLK-CYC-mediated transcription in a complex manner that is balanced between stimulation and inhibition via adjusting the accessibility of both positive and negative factors to the chromatin. This would explain the discrepancy in the observed differences in circadian gene expression in the two classes of *brm* knockdown.

### Non-catalytic function of BRM negatively affects CLK binding to the *per* promoter

**Fig 4:**
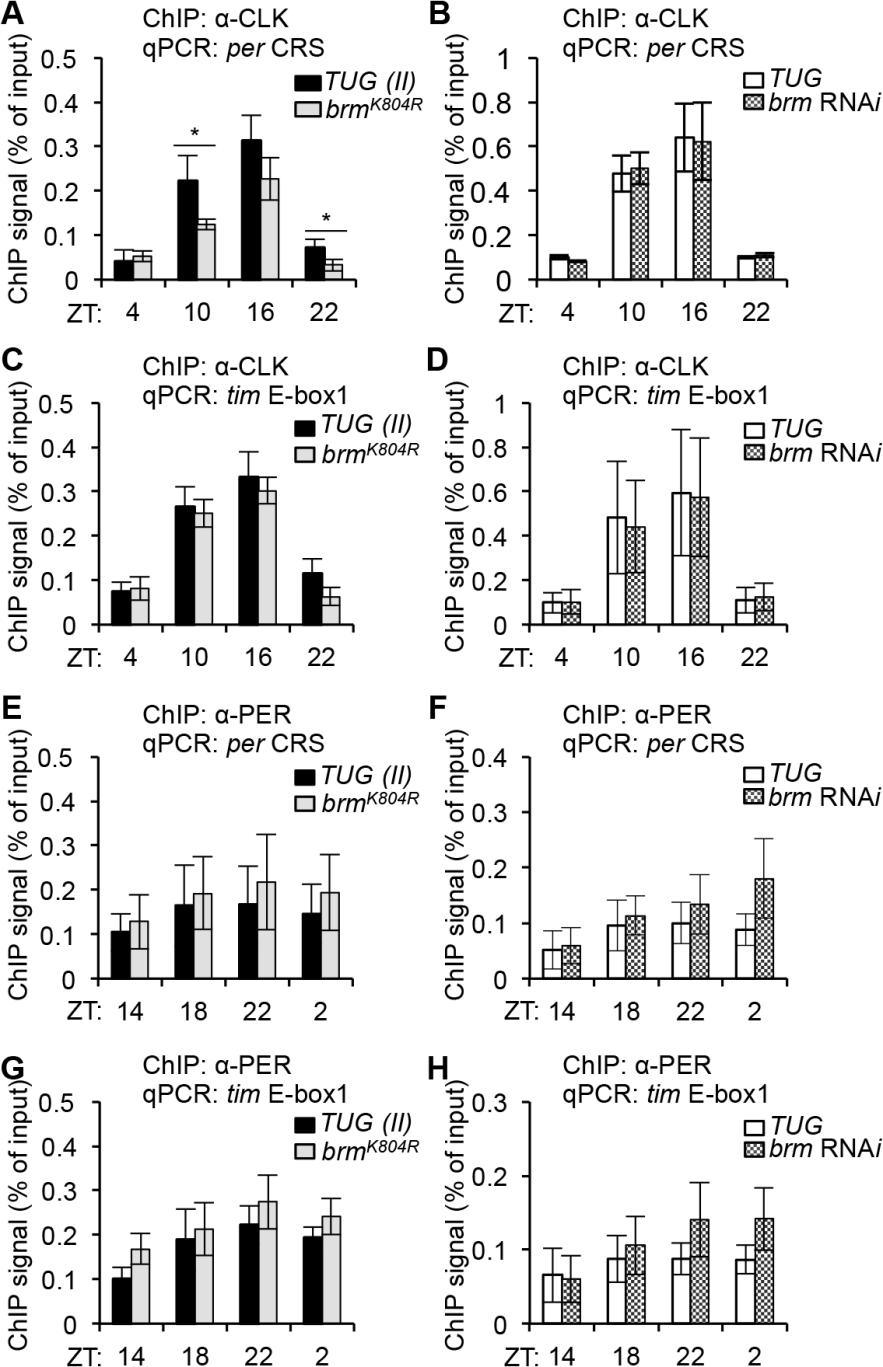
The non-catalytic function of BRM negatively impacts CLK binding to the *per* promoter. ChIP assays detecting CLK binding at the *(A-B) per* CRS and *(C-D) tim* E-box 1 in flies expressing (*A* and *C*) catalytically-inactive *brm* ^*K804R*^ and (*B* and *D*) *brm* RNA*i* (v37720) in *tim*-expressing cells as compared to control *TUG(II)* and *TUG* flies. Corresponding ChIP assays detecting PER binding at the (*E-F*) *per* CRS and (*G-H*) *tim* E-box 1 in flies expressing (*E* and *G*) *brm* ^*K804R*^ and (*F* and *H*) *brm* RNA*i* as compared to control *TUG(II)* and *TUG* flies. Results shown are from at least three biological ChIP replicates, with technical triplicates performed during qPCR for each biological replicate. Error bars = SEM of biological replicates. Asterisks denote significant difference between control and *brm* mutant at indicated time points (P < 0.05).

Since relaxed chromatin structure has been associated with being more permissive to transcription factor binding (reviewed in [58]), and we observed changes in clock gene expression in the two different classes of *brm* knockdown flies, albeit in opposite direction, we next examined whether CLK (activator) and PER (repressor) binding to *per* and *tim* promoters was affected in flies expressing either *brm* RNA*i* or the *brm* ^*K804R*^ transgene using ChIP-qPCR (S7 Fig), and whether differential CLK and/or PER binding contributed to the discrepancies in gene expression levels observed in flies expressing *brm* RNA*i* and *brm* ^*K804R*^. CLK binding was not expected to be affected by the decrease in nucleosome occupancy, since in mammals, CLOCK:BMAL1 have been shown to bind to nucleosome-bound DNA [28]. In control *TUG* driver lines, we observed the rhythmic binding of CLK to the *per* and *tim* promoters that has previously been reported (Fig 4A–4D) [51]. Indeed, we did not see an increase in CLK occupancy in flies expressing *brm* RNA*i* nor *brm* ^*K804R*^ despite the decrease in nucleosome density (Fig 4A–4D). This suggests that the *brm* RNA*i*-induced increase in CLK target gene expression (Fig 3A and 3B) is due to mechanisms downstream of CLK binding, likely because of the more open chromatin. However, we observed a significant decrease in CLK binding on the *per* promoter in flies expressing BRM^K804R^, especially during the daily upswing in gene expression (Fig 4A), suggesting that the non-catalytic activity retained by BRM^K804R^ exhibits inhibitory effects on CLK binding to its target genes that becomes more prominent with decreased nucleosome density. As PER does not generally enter the nucleus until ~ZT18 [59], the negative effect of BRM^K804R^ on CLK binding to the *per* promoter is expected to be independent of PER repression. It is interesting to note that we did not see a significant decrease in CLK binding on the *tim* promoter in flies expressing *brm* ^*K804R*^ as we did for the *per* promoter (Fig 4C). This suggests that while *per* and *tim* are both activated by CLK, binding of CLK to the *tim* promoter may be more conducive, and therefore less sensitive to the inhibitory effects introduced by BRM^K804R^ (Fig 4A and 4C). Supporting this, there is clear evidence that the transcription rate of *tim* is higher than *per* with higher amplitude cycling [60]. This could be partially due to the presence of additional non-canonical E-boxes in the *tim* promoter [61].

Another possible factor contributing to the decrease in CLK binding at the *per* promoter is that overall CLK levels may be reduced in flies expressing *brm* ^*K804R*^. Since *Clk* expression is regulated by proteins encoded by two CLK-activated targets, *vri* and *pdp1ε*, and we have already shown that expression of these genes are reduced in flies expressing *brm* ^*K804R*^ (S5B Fig), it is possible that *Clk* transcription is affected as well, resulting in decreased CLK expression. Indeed, gene expression analysis by qPCR (S8 Fig) and immunoblotting (S9A and S9B Fig) confirmed that both *Clk* mRNA and CLK proteins are decreased in the *brm* ^*K804R*^ mutant. The large decrease in CLK protein compared to the relatively modest decrease in *Clk* mRNA in flies expressing *brm* ^*K804R*^ suggests that the non-catalytic activity of BRM could affect CLK stability. Although, if that is indeed true, we would expect to see a decrease in CLK binding at the *tim* promoter as well. Overall, our results suggest that the observed decrease in CLK target gene expression in flies expressing *brm* ^*K804R*^ may partly be a consequence of reduced CLK binding to their promoters due to lower CLK levels. But since *tim* expression decreases in flies expressing *brm* ^*K804R*^ despite similar levels of CLK binding as compared to control flies (Fig 4C), it is likely that the non-catalytic function of BRM negatively impacts CLK target gene expression through additional mechanisms outside of its effects on CLK binding.

We also assayed the levels of *Clk* mRNA and protein in flies expressing *brm* RNA*i* and found that as with *per* and *tim*, both mRNA and protein levels were elevated (S8 Fig and S9C and S9D Fig). Although CLK levels are elevated in flies expressing *brm* RNA*i*, no increase in CLK binding at the *per* and *tim* promoters was observed (Fig 4B and 4D), suggesting that total CLK levels alone cannot fully account for the observed changes in CLK binding to target promoters. Nevertheless, lower CLK levels might be rate-limiting with respect to transcription under certain circumstances, e.g. *per* transcription in flies expressing *brm* ^*K804R*^ (Figs 3C and 4A).

In addition to changes in CLK binding, alterations in PER binding to CLK target gene promoters in flies expressing either *brm* RNA*i* or *brm* ^*K804R*^ could also contribute to the observed changes in gene expression. We therefore assayed PER binding to *per* and *tim* promoters in both *brm* mutants using ChIP-qPCR, but did not detect significant changes in PER binding to either promoter (Fig 4E–4H). The level of PER binding to *per* and *tim* promoters also did not appear to be sensitive to the differential amount of PER resulting from higher *per* expression in *brm* RNA*i* flies and decreased *per* expression in flies expressing the *brm* ^*K804R*^ transgene (S9A and S9B Fig). We therefore conclude that the level of PER binding to *per* and *tim* promoters does not appear to be a significant factor that could explain the observed differences in *per/tim* mRNA levels between flies expressing *brm* RNA*i* and *brm* ^*K804R*^.

Thus, the combined results suggest that BRM directly suppresses expression at the *per* and *tim* promoters by increasing nucleosome occupancy and in some still undefined manner inhibiting transcription in a catalytically independent manner. Of course, due to the interconnected feedback loops, the effects of BRM on the levels of positively (e.g., PDP1ε) and negatively (e.g., PER, VRI) acting factors could in turn contribute to changes in the levels and/or activity of CLK, further modulating clock gene expression. However, the fact that clock gene expression levels do not correlate very tightly with either CLK or PER levels, suggests that the main effects of BRM on clock gene expression are direct.

### The chromatin landscape established by BRM plays a role in regulating RNAPII dynamics

Chromatin remodelers, such as the *Brahma* complex, are known to influence transcriptional regulation at the level of chromatin structure as more open chromatin is generally believed to be associated with less physical blockage for RNAPII. In addition, the *Brahma* complex was shown to interact with RNAPII and regulate its activity through transient stalling downstream of the transcription start site (TSS) [62]. RNAPII stalling or pausing has been shown to heavily regulate the expression of many eukaryotic genes and represents an additional step in transcriptional regulation beyond RNAPII recruitment (reviewed in [63]). Pausing for most genes has been observed around 30 to 100 downstream of the TSS [64], but pausing even at 1.5 kb into the coding region has been observed [62]. To obtain a more comprehensive understanding of the progression of RNAPII at the *per* and *tim* loci, two additional regions along the genes were analyzed by ChIP-qPCR in addition to the E-boxes initially identified as BRM binding sites (S10 Fig). The TSS as well as a region within the gene body (coding region) was analyzed for each gene. The RNAPII antibody used for our ChIP analysis recognizes RNAPII with phosphorylated CTD repeat (serine 2 and serine 5) with preference to serine 5, therefore it is expected to detect initiated and transiently paused RNAPII and to a lesser extent, elongation-competent RNAPII [27, 65].

**Fig 5:**
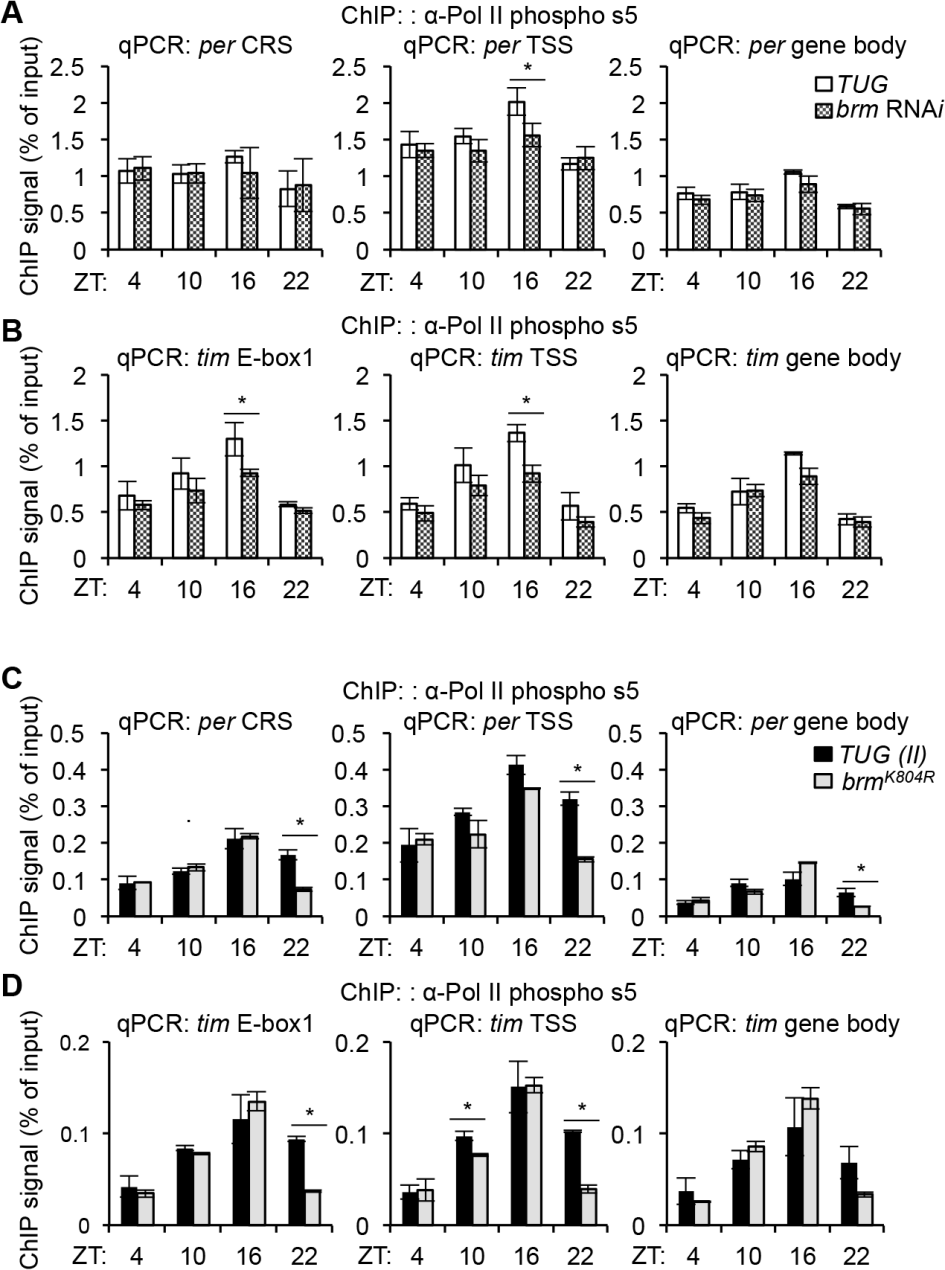
BRM-established chromatin landscape plays a role in regulating RNAPII dynamics. ChIP assays to detect RNAPII occupancy at three regions: CLK binding site (*per* CRS and *tim* E-box 1), TSS (Transcription Start Site), and gene body along the *(A) per and (B) tim* loci in flies expressing *brm* RNA*i* (v37720) (grey bars) as compared to control *TUG* flies (white bars). *(C and D)* The same RNAPII ChIP assay was performed using flies expressing *brm* ^*K804R*^ (grey bars) compared to control *TUG(II)* flies (black bars). Flies were collected at the four indicated time points (ZT). Results shown are from three biological replicates, with technical triplicates performed during the qPCR step for each biological replicate. Error bars = SEM of biological replicates. Asterisks denote significant difference between control and *brm* mutant at indicated time points (P < 0.05).

When measuring RNAPII occupancy in control flies, we observed constitutive binding at the *per* CRS (Fig 5A, left panel), consistent with previously reported findings [26], as well as an increased occupancy at the TSS, a signature of possible RNAPII transient stalling (Fig 5A, middle panel). This increase in RNAPII occupancy at the *per* TSS was particularly prominent at ZT16, possibly for fine-tuning *per* RNA transcript abundance during a time in the daily cycle when CLK-CYC-mediated transcription is high. The overall RNAPII occupancy throughout a daily cycle decreased as we examined the downstream region within the *per* gene body (Fig 5A, right panel), although the RNAPII we detected may be more reflective of initiated or transiently paused RNAPII. In flies expressing *brm* RNA*i*, there was a significant drop in RNAPII occupancy at ZT16 as compared to the parental control at ZT16 (Fig 5A, middle panel), suggesting that the extent of RNAPII pausing at the *per* TSS was diminished due to decreased nucleosome density when *brm* was knocked down, which could contribute to the increase in *per* expression observed in these flies.

At the *tim* locus, we did not observe an increase in RNAPII occupancy at the TSS as compared to the *tim* E-box 1 or the gene body when comparing RNAPII occupancy at respective time points (Fig 5B, compare left, middle, and right panels), indicating that transient RNAPII pausing may not play as an important role in *tim* transcription. However, we observed prominent rhythmic RNAPII recruitment that coincides with the gene activation phase (higher at ZT10 and ZT16) in all gene regions (Fig 5B), consistent with earlier findings [26]. Knocking down *brm* by RNA*i* significantly decreased RNAPII occupancy at *tim* E-box 1 and the TSS at ZT16 (Fig 5B, left and middle panel) and this difference appeared less pronounced in the gene body (Fig 5B, right panel). The decrease in RNAPII occupancy at the *tim* E-box and the TSS is also present earlier in the gene activation phase at ZT10, though not statistically significant. It is intriguing that at ZT16 in *brm* RNAi flies, both *per* and *tim* exhibit strong reductions in initiated or paused RNAPII occupancy at their respective TSS, suggesting a common mechanism contributing to their increased expression levels (Fig 3). Nonetheless, our data supports previous findings [26] that *per* and *tim* transcription, although both activated by CLK-CYC, are regulated through different mechanisms at the chromatin level: *per* appears to be regulated at the level of RNAPII pausing, perhaps in addition to RNAPII recruitment, whereas rhythmic RNAPII recruitment appears to play a more important role in regulating *tim* transcription. Future RNAPII ChIP-seq studies can be performed to provide higher resolution insight to the extent of RNAPII stalling on circadian promoters.

Besides regulating nucleosome density through its catalytic activity, non-catalytic activities of BRM could also influence RNAPII activity. We therefore assayed RNAPII occupancy and activity at the *per* and *tim* loci in flies expressing *brm* ^*K804R*^. Interestingly, in the *TUG(II)* control flies used for these experiments, we observed weak (but not significant) cycling of RNAPII recruitment on *per* (Fig 5C), which was not as apparent in the *TUG* control flies used for comparison with flies expressing *brm* RNA*i* (Fig 5A and 5B). RNAPII recruitment for *tim* remains rhythmic as observed earlier (Fig 5D). This suggests that although it is clear RNAPII pausing regulates *per* more than *tim* transcription, temporal changes in RNAPII recruitment may still contribute to the cyclical regulation of *per* transcription.

When comparing RNAPII occupancy in control and flies carrying the *brm* ^*K804R*^ mutation, we did not observe a significant decrease in RNAPII occupancy at the *per* TSS (Fig 5C, middle panel), which was very prominent at ZT16 in flies expressing *brm* RNA*i* (Fig 5A, middle panel). Similarly, the significant decrease in RNAPII occupancy at the *tim* promoter (*tim* E-box 1 and TSS) at ZT16 when *brm* was knocked down by RNA*i* (Fig 5B, left and middle panels) was also absent in flies expressing the *brm* ^*K804R*^ transgene (Fig 5D, left and middle panels). The fact that the occupancy of initiated and/or paused RNAPII did not show significant changes in flies expressing *brm* ^*K804R*^ in a nucleosome depleted chromatin landscape, which should be more permissive to RNAPII elongation, supports our hypothesis that the non-catalytic function of BRM^K804R^ have a repressive effect on transcription. However, we should point out that although insignificant, flies expressing *brm* ^*K804R*^ showed some decrease in RNAPII occupancy in the *per* TSS at ZT 10 and 16 (Fig 5C) and the *tim* promoter in ZT10 (Fig 5D), similar to the trend observed in *brm* RNA*i* flies (Fig 5A and 5B). We therefore suggest that the contribution of the catalytic activity in modulating RNAPII dynamics in circadian genes cannot be ruled out at this point. Curiously, we noticed a consistent significant decrease in RNAPII occupancy in the *brm* ^*K804R*^ mutant at ZT22 in all regions sampled within *per* and *tim* (Fig 5C and 5D), suggesting that the non-catalytic function of BRM may promote the removal of RNAPII at ZT22 at the end of the transcription cycle. Further investigation is required to understand the significance of the decreased RNAPII occupancy at ZT22.

### BRM does not affect alternative splicing of *per* transcripts

The *Brahma* complex has previously been implicated in pre-mRNA splicing regulation of developmental genes [54, 66]. Previous studies have also identified that in *Drosophila*, *per* undergoes alternative splicing and there are two transcripts that occur naturally, which affect accumulation of *per* mRNA [67–68]. These two transcripts differ only by the presence or absence of an alternative intron in the 3’ untranslated region (UTR) and increased *per* splicing leads to higher levels of *per* mRNA. We therefore sought to investigate if BRM affects the alternative splicing of *per* in the 3’ UTR, and test the hypothesis that knocking down *brm* could lead to changes in splicing efficiency and contribute to the change in *per* mRNA levels observed in flies expressing *brm* RNA*i*. Using primers that flank the previously identified alternatively spliced intron in the 3’ UTR of *per* [68], we measured the relative abundances of spliced and unspliced transcripts using semi-quantitative PCR and quantified the unspliced transcripts relative to total *per* mRNA levels (total of spliced and unspliced transcripts). Both unspliced and spliced *per* levels were normalized using *cbp20* as an internal control. While we were able to identify both alternatively spliced transcripts, we did not observe a significant difference in the splicing efficiency in flies expressing *brm* RNA*i* knockdown as compared to control flies (S11 Fig), showing that BRM is unlikely to regulate *per* levels via this splicing event.

## Discussion

**Fig 6:**
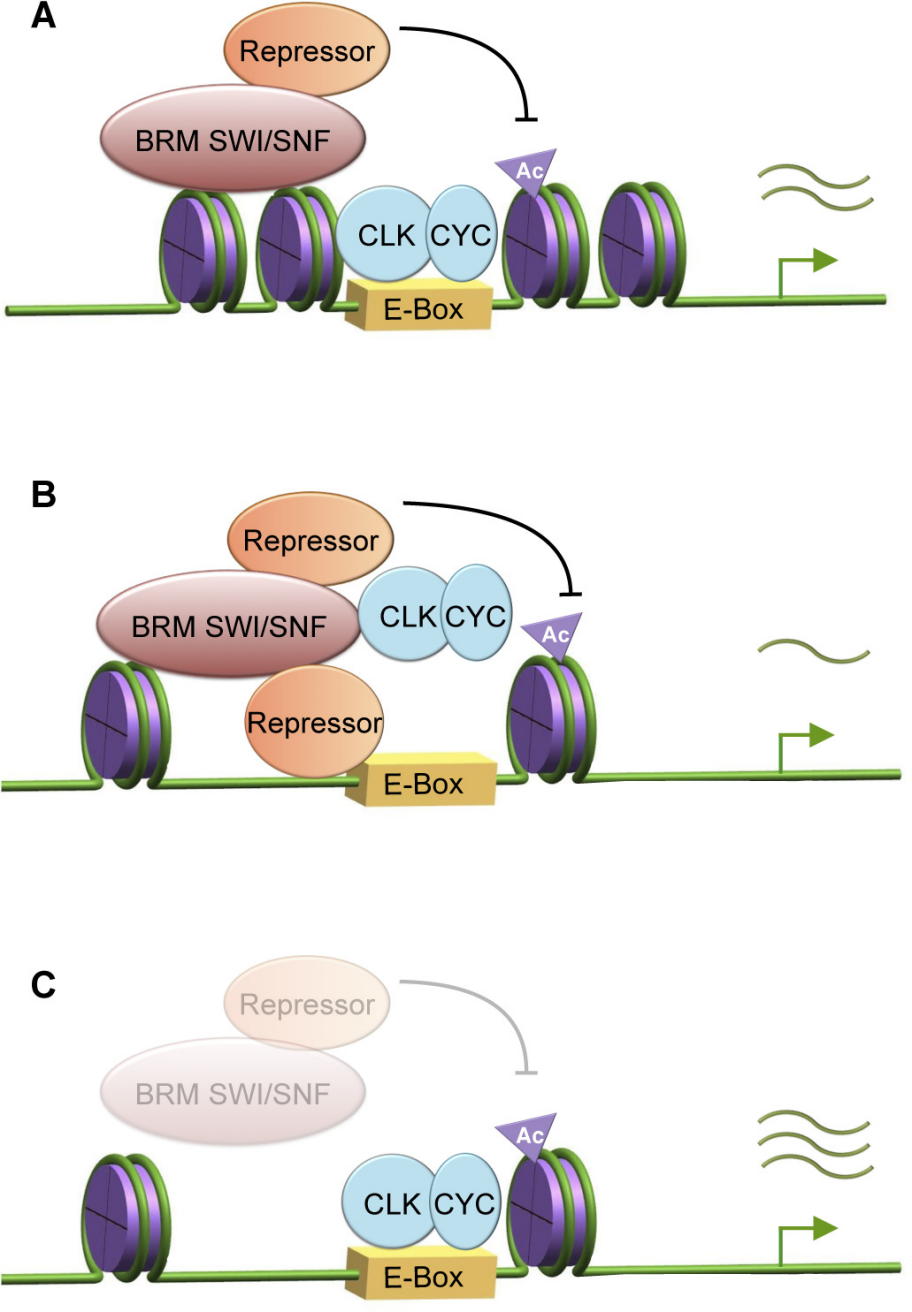
BRM exhibits both catalytic and non-catalytic roles in fine-tuning circadian transcription. *(A)* In wild type flies, BRM remodels chromatin at *per* and *tim* promoters and maintains nucleosome density to fine-tune CLK dependent transcription. Chromatin compaction during active transcription phase appears to be necessary for transient RNAPII stalling and may prevent excessive recruitment of BRM-bound repressive complexes, e.g. HDACs that can modulate active histone marks. *(B)* Incorporation of catalytically-inactive BRM^*K804R*^ into endogenous *Brm* complexes results in a decrease in nucleosome density. The more open chromatin then augments the non-catalytic function (including scaffolding properties) of BRM^*K804R*^ that may be involved in recruiting repressive complexes that negatively regulate CLK binding at the *per* and *tim* promoter. Possible recruitment of pausing factors by BRM is not affected, resulting in wild type levels of RNAPII stalling. BRM^*K804R*^ may also negatively affect the level of *Clk* expression by decreasing transcriptional activities at the *Clk* locus (not shown in model). Summation of these effects leads to a decrease in CLK target gene expression. *(C)* In flies expressing *brm* RNA*i*, knockdown of *brm* expression results in decreases in both catalytic and scaffolding activities, leading to loss of nucleosome density, impaired RNAPII stalling at the TSS during peak of transcription output (ZT16), as well as reduction in recruitment of BRM-bound repressive complexes. The consequence is an increase in CLK target gene expression.

Results accumulated from investigations across multiple organisms now enable the correlation between the progression of rhythmic circadian gene expression and the timing of a number of regulatory events that take place at the transcriptional level (reviewed in [1, 3, 24, 25]). These interconnected regulatory events include the rhythmic recruitment of transcriptional activators and repressors, changes occurring at the chromatin resulting from recruitment of histone modifiers, as well as oscillating RNAPII recruitment and/or elongating activity. In comparison, except in the case of the fungus *Neurospora crassa* [32–35], less is known about the correlation between circadian transcription and nucleosome dynamics. As nucleosome density, phasing, positioning, and composition all influence gene transcription [69–70], it is logical to predict that nucleosome dynamics could play an important role in regulating the circadian transcriptome. Here, we have identified the *Brahma* (SWI/SNF) chromatin-remodeling complex as a regulator of nucleosome dynamics at key circadian clock genes in *Drosophila* and investigated its role in maintaining a robust circadian oscillator. We demonstrated that BRM exerts both catalytic and non-catalytic activity on CLK target gene transcription, and the proper balance of these activities is critical for clock function (Fig 6).

### A model describing the intricate balance between catalytic and non-catalytic functions of BRM to fine-tune circadian transcription

The comparison between flies expressing the catalytically-inactive BRM^K804R^ protein and *brm* RNA*i* has given us the opportunity to tease apart the catalytic and non-catalytic roles of *brm* in circadian transcription. Since flies with two distinct genetic manipulations to reduce the endogenous influence of BRM in clock cells both exhibit a reduction in nucleosome density, it was initially surprising that they showed opposite phenotypes with respect to changes in steady state CLK target gene expression (Fig 3A–3D). In particular, our results showing that flies expressing *brm* ^*K804R*^ have lower expression levels of CLK-dependent clock gene targets as compared to control flies suggests that the interactions of BRM to other proteins, which is retained in BRM^K804R^, negatively regulates output during the active transcription phase. Since we showed that BRM interacts with CLK, it is possible that BRM^K804R^ is working in a dominant negative manner to limit CLK levels, binding and/or activity to negatively regulate CLK target gene expression. Consistent with this idea, the abundance of CLK was lower in flies expressing BRM^K804R^ and the binding of CLK to the *per* promoter was also reduced (Figs 4A and S9A and S9B). However, BRM^K804R^ had little to no effect on the levels of CLK binding to the *tim* promoter (Fig 4C), suggesting that effects on CLK binding to chromatin cannot fully explain the inhibitory effect of BRM on all clock target gene expression.

Indeed, there is extensive evidence indicating BRM interacts with many transcriptional regulators, including repressors, as well as RNAPII and associated factors [62, 71–75]. We postulate that BRM recruits repressive complexes to negatively regulate CLK binding and/or restrain CLK activity during the active transcription phase, and these interactions are precisely balanced by the catalytic function to increase nucleosome density to prevent over-accumulation of repressive complexes or histone marks (Fig 6A). Possible candidates of repressive complexes will be discussed below. With the intact scaffolding function but defective BRM ATPase activity failing to compact chromatin in flies expressing *brm* ^*K804R*^, the more open chromatin could augment the non-catalytic function of BRM^K804R^ and lead to over-recruitment of repressive complexes or proteins that reduce CLK stability, resulting in decreased circadian gene transcription (Fig 3C and 3D, Fig 6B). On the other hand, since both the catalytic and non-catalytic functions of BRM are reduced in *brm* RNA*i* flies, the repressive complexes normally recruited by BRM would be reduced, thereby leading to an increase in gene expression. Moreover, transcription activators or machineries independent of BRM non-catalytic activity could be more abundant due to the open chromatin, also contributing to the increase in CLK target gene transcription (Fig 3A and 3B, Fig 6C).

Since the timing and extent of transcriptional repression at the CLK targets that were tested remained unchanged in flies expressing either *brm* RNA*i* or *brm* ^*K804R*^ (Fig 3A–3D), we suggest that BRM preferentially functions to control the rate of transcription during the daily upswing in CLK-dependent clock gene expression with minimal effects on the initiation and/or maintenance of transcriptional repression, a role likely played by a different class of chromatin remodeler. Furthermore, the fact that the interaction of CLK and BRM as assayed by co-IP in flies appeared to peak at times of active transcription lends further support to a role for BRM in modulating CLK-dependent transcription (S1 Fig). Co-IP in flies also showed that BRM interacts with TIM in the late evening. It is possible that TIM might be involved in terminating BRM function at CLK target genes. Future analysis will be necessary to test this hypothesis.

Our results indicate that the role of BRM on *Drosophila* CLK target genes is different from that of the orthologous SWI/SNF complex in *Neurospora*, in which it was shown to open up the chromatin at the *frq* locus to facilitate transcriptional activation [35]. In contrast to SWI/SNF in *Neurospora*, the ATP-dependent nucleosome remodeling activity of BRM on CLK targets increases nucleosome occupancy, especially during times in a daily cycle when transcriptional output from these genes are peaking. Although at a low amplitude, circadian cycles of BRM-mediated chromatin compaction and relaxation at these promoters can be observed with peak compaction coinciding with times of active transcription even in wild type control flies, and these rhythms were abolished in flies in which BRM catalytic activity was reduced (Fig 3E–3H). In support of our findings indicating an overall repressive role of BRM in limiting transcription output of CLK target gene promoters and its catalytic function to increase nucleosome density, recent genome-wide studies examining BRM function in *D*. *melanogaster* larval tissues [76] and primary mouse cells [55] showed that knocking down BRM led to widespread disruption of nucleosome organization, with a bias towards a decrease in nucleosome density, especially at promoters and peri-TSS regions. Furthermore, although the relationship between loss of nucleosome density upon BRM knockdown and changes in gene transcription is highly variable, more genes appeared to be significantly upregulated than downregulated, thus corresponding with our findings in *Drosophila* CLK target genes. The variable outcome from loss of nucleosome density could be explained by the specificity of BRM to recruit either activators or repressors on a gene-by-gene basis. Our model proposes a complex scheme in which the catalytic activity of chromatin remodelers such as BRM can function like a rheostat to adjust the non-catalytic function to control factor recruitment and the rate of transcription in an intricate manner.

### Accumulating evidence indicates BRM can recruit repressive complexes

Over the years, BRM has been identified to play a role in both transcriptional activation and repression [41, 56, 57], and it has become increasingly evident that whether it acts as an activator or repressor highly depends on the proteins recruited through its non-catalytic activity. Based on our results, BRM appears to recruit repressive complexes to negatively regulate CLK activity and binding to target genes. In many genes including clock genes, dynamic deacetylation of histones in the promoter region has been implicated in the repression of gene expression (reviewed in [24–25]). Histone deacetylation is catalyzed by a large class of histone deacetylases (HDACs), and many HDACs and other epigenetic modifiers have been found to be under circadian control as well as having direct interactions with clock proteins. The transcriptionally repressive Sin3-HDAC complex, which is an evolutionarily conserved protein complex that includes HDAC1 and HDAC2 [77], has been found to co-precipitate with PER complexes and aid in the repression of circadian transcription [78]. BRG1 (mammalian homolog of *Drosophila* BRM) and other components of the SWI/SNF complex have also been found to co-immunoprecipitate with components of Sin3-HDAC [72–74], showing precedence in direct association of BRM (SWI/SNF) with proteins involved in histone deacetylation and gene repression. In addition to interacting with HDAC1 and HDAC2-containing complexes, BRG1 and other *Brahma* complex related proteins have also been observed to associate with a corepressor complex N-CoR-1, which contains HDAC3 [71], The accumulating evidence of interactions of the BRM complex with repressive factors provides support to our model in which we postulate that BRM is involved in limiting CLK target gene output.

### BRM affects RNAPII dynamics at circadian genes

Changes in nucleosome occupancy have been known to regulate transcription factor binding [70] and differential transcription factor binding has the potential to affect transcriptional output. We therefore sought to examine CLK and PER binding at *per* and *tim* promoters in the two different types of *brm* knockdown (Fig 4). Although we found a decrease in CLK binding in flies expressing *brm* ^*K804R*^ that could partly explain the reduced transcriptional output at CLK target genes (Fig 4A), we found no significant differences in CLK and PER binding in flies expressing *brm* RNA*i* even though they showed a large increase in transcriptional output at CLK target genes (Fig 4B, 4D, 4F and 4H). We therefore explored whether changes in chromatin organization in these flies can influence RNAPII dynamics [79–80] to increase CLK target gene expression. Using RNAPII ChIP, we found that the increased nucleosome density mediated by BRM, at times when CLK-mediated transcription is peaking (i.e., ZT16), appears to be associated with an increase in transient RNAPII stalling near the *per* TSS (Fig 5A and 5C). This was not observed in the case of the *tim* TSS as RNAPII dynamics at *tim* appears to be more heavily regulated through RNAPII recruitment, rather than transient stalling (Fig 5B and 5D). The lack of transient stalling at the *per* promoter observed in flies expressing *brm* RNA*i* could be due to the decrease in nucleosome density and increase in RNAPII elongation into the gene body resulting in higher overall transcription output (Fig 5A). This scenario is supported by the fact that there is also a general reduction in RNAPII occupancy in the *tim* promoter and TSS (Fig 5B) in the *brm* RNAi flies that could also lead to increased mRNA output. Originally, we had anticipated that we might observe an increase in RNAPII occupancy in the gene body that corresponds with a decrease in RNAPII occupancy at the *per/tim* E-boxes and TSS in flies expressing *brm* RNA*i*, which would reflect the increased transition of initiated/paused RNAPII at the TSS into active elongation in the gene body. This was not observed, and could be due to the fact that the RNAPII antibody we used has a higher affinity for initiated/paused RNAPII, rather than elongating RNAPII.

In addition to changes in nucleosome density, it is worthy to point out that there could be additional mechanisms that regulate the observed transient stalling at the *per* TSS, including the recruitment of pausing factors via the non-catalytic function still retained in BRM^K804R^. In *Drosophila*, it has been shown that BRM and a known interactor SAYP facilitates the formation of nucleosome-dense regions that acts as barriers to RNAPII, leading to stalling during the repressive phase of transcription [61]. Elimination of SAYP and BRM results in loss of this nucleosomal barrier, thereby releasing RNAPII and leading to an increase in transcription. These findings corroborate with our observation of the lack of RNAPII stalling at the *per* promoter in *brm* RNA*i* mutants. Stalling could be unaffected in flies expressing *brm* ^*K804R*^ because BRM^K804R^ still retains the ability to interact with pausing factors such as SAYP.

Altogether, our results indicate that BRM functions to fine-tune CLK-CYC-mediated expression of core clock genes by acting as a braking mechanism when transcriptional activation is in full effect. It appears that *Drosophila* circadian transcription during the active phase may be operating under a largely restrictive chromatin landscape, and this mechanism may be important in maintaining precise levels of cyclical gene expression. Our data suggests that this fine-tuning occurs through the coupling of the catalytic and non-catalytic functions of BRM, thereby generating a balanced chromatin landscape for transcription factor binding, transient RNAPII stalling, and possible scaffolding interactions with histone modifiers such as HDACs. Future experiments will aim at clarifying the interaction between the *Brahma* complex and histone modifiers, as well as the possible role of *brm* in regulating the expression of clock genes such as *Clk* that cycle in anti-phase to those that are direct targets of CLK-CYC.

## Materials and Methods

### Generation of fly lines and genetic crosses

Targeted RNA*i* knock down of *Brahma (Brm)* subunits in circadian clock neurons was achieved using the UAS/GAL4 system [81]. To knock down individual subunits, virgin female flies from *UAS*-RNA*i* responder lines were crossed to males from driver line *w; UAS-dicer2; tim-UAS-GAL4* (referred to as *TUG*) [45] to achieve knockdown in *tim*-expressing neurons. Up to three independent *UAS*-RNA*i* responder lines targeting each gene were used. *UAS-dicer2* was included to increase the efficiency of RNA interference. Male progenies of the crosses were then assayed for locomotor activity. Both male and female progenies were used for RNA and protein assays. Targeted knockdown of *brm* in PDF neurons (a subset of clock neurons) was achieved with the use of a *pdf-GAL4* driver line (obtained from P. Hardin). To rule out developmental effects caused by *brm* RNA*i* leading to clock defects, we utilized the temperature sensitive GAL80 mutant (Bloomington Drosophila Stock center stock number 7108) to inhibit GAL4 expression during development. Progenies from crosses were placed into incubators set at 18°C to inhibit expression of dsRNA during development and transferred to 29°C to relieve the GAL4 inhibition three days before behavioral assays. To generate *brm* overexpressing flies, 3XFLAG-6XHis-*brm* was cloned into pUAST vector (Addgene). This plasmid was injected into *w* ^*1118*^ embryos by Genetic Services, Inc (Sudbury, MA). Transgenic flies carrying the *UAS*-FLAG-*brm* transgene were then crossed to *w; tim-(UAS)-GAL4* driver line (referred to as *TUG(II*)) to obtain expression of FLAG-*brm* in clock neurons.

### Locomotor activity assays

Male flies around 3 to 5 days old were subjected to locomotor activity assays using the Drosophila Activity Monitoring System (DAMS) (Trikinetics, Inc.). Flies were entrained for four days at 12 hr light:12 hr dark (LD) conditions at 25°C before their free-running behavioral rhythms were assessed in total darkness (DD) for seven days. Fly activity monitoring using DAMS and data analysis using FaasX were as previously described [82]. Behavioral assays to rule out developmental effects of *brm* RNA*i* were performed at 29°C.

### Plasmid construction for co-immunoprecipitation in *Drosophila* S2 cells

For co-immunoprecipitation (co-IP) in *Drosophila* S2 cells, *brm* cDNA clones were obtained from *Drosophila* Genomics Resource Center (DGRC) and processed according to the stock center protocol. The *brm* ORF was amplified from cDNA reverse transcribed from total RNA extracted from fly heads without the stop codon using PCR and the PCR product was subcloned into pAc-3XFLAG-6XHis [10] such that the ORF of *brm* is in frame and located at the N terminal end of the FLAG epitope. The plasmids expressing pAc-*per*-V5-His and pAc-*tim-*3HA were previously described [83]. The pAc-*clk-*V5-His plasmid was generated by restriction digesting *clk* from pMT-HA-*clk-*V5 [84] and subcloned into pAc-V5-His vector.

### Antibodies

We generated α-PER (GP5620) by using PCR to amplify *per* cDNA sequences that encode amino acids 232 to 599 and cloned the PCR fragment upstream of sequences that encode a polyhistidine stretch (His) in the expression vector pET28b (Novagen). Fusion protein expression, purification, and antibody production were performed as previously described [85]. Antibodies to TIM (R3) used in our experiments were as described [85]. Commercially available antibodies were purchased for CLK (Santa Cruz Biotechnology H3107), FLAG (Sigma F3165), histone H3 (Abcam ab1791), and RNAPII (Abcam ab5408). The RNAPII antibody used for ChIP recognizes RNAPII with phosphorylated CTD repeat (Serine 2 and Serine 5) with preference to Serine 5. Antibody dilutions are listed below.

### Co-immunoprecipitation in *Drosophila* S2 cells

To perform co-IP assays, 3 x 10^6^ S2 cells were transiently transfected with pAc-*brm-*3XFLAG-6XHis in combination with pAc-*per*-V5-His, pAc-*tim-*3HA, or pAc-*clk-*V5-His using the Qiagen Effectene Transfection reagent following manufacturer’s protocol. 48 hours after transfection, cells were harvested for protein extraction using M-RIPA buffer (20 mM Tris-HCl at pH 7.5, 150 mM NaCl, 1 mM EDTA, 10% glycerol, 1% Triton X-100, 0.4% DOC, 0.1% SDS, 0.5 mM PMSF, 10 μg/μl aprotinin, 10 μg/μl leupeptin, 2 μg/μl pepstatin A, 25 mM NaF, and 1x Roche protease inhibitor). Protein extracts were quantified and either directly analyzed by immunoblotting (input lysate) or aliquoted for IP. BRM IP samples were incubated with α-FLAG M2 affinity gel (Sigma). In addition, reciprocal IP and negative control IP samples were incubated with either α-V5 agarose beads (Sigma) or α-HA agarose (Sigma) depending on the clock protein (PER, TIM, or CLK) that is coexpressed with BRM. Following 4 hours of incubation at 4°C, beads were washed a total of three times (10 minutes per wash) in a M-RIPA buffer with increased salt (300 mM NaCl). Immune complexes were eluted from beads by adding 2X SDS-PAGE sample buffer and resolved using SDS-PAGE. For protein visualization, α-FLAG (1:7000) was used to detect BRM-FLAG, α-V5 (1:5000) was used to detect PER and CLK. Secondary antibody for α-FLAG and α-V5 detection was α-mouse IgG-HRP at 1:2000 (GE Healthcare). α-HA (1:1000) was used to detect TIM with secondary antibody α-rat IgG at 1:1000 (GE Healthcare).

### Western blotting of fly protein extracts

Flies were entrained for three full days in 12 hr light:12 hr dark (LD) conditions at 25°C and collected on the fourth day at the indicated time points (ZT) and frozen immediately on dry ice. Heads were separated using frozen metal sieves and homogenized in 3x volume of RBS buffer (20 mM HEPES at pH 7.5, 50 mM KCl, 10% glycerol, 2 mM EDTA, 1 mM DTT, 1% Triton X-100, 0.4% NP-40, 10 μg/mL aprotinin, 5 μg/mL leupeptin, 1 μg/mL pepstatin A, 0.5 mM PMSF, 1X PhoStop (Roche)) [46, 51]. Homogenate was sonicated using a Fisher Scientific sonicator for five seconds and repeated five times with 10-second pauses in between. Samples were spun down at 14,000 rpm for 15 minutes at 4°C to remove cell debris. Supernatant was collected, transferred to new tubes, and spun down again for 10 minutes at 14,000 rpm at 4°C. Supernatant was collected and protein levels were quantified using a spectrophotometer (Eppendorf). Proteins were resolved by SDS-PAGE (Criterion 5% gels, Bio-Rad), transferred to nitrocellulose membranes (Bio-Rad) and incubated in 5% blocking solution (Bio-Rad) in 1XTBST with α-PER (GP5620) (1:2000), α-TIM R3 (1:2000), or α-CLK (Santa Cruz) (1:1000). Membranes were imaged and protein levels were quantified using the ChemiDoc MP system with Image Lab software (Bio-Rad).

### Co-immunoprecipitation using fly protein extracts

Transgenic flies expressing BRM fused to FLAG epitope tags were entrained in 12 hr light:12 hr dark (LD) conditions at 25°C for three days and collected on the fourth day. Fly head collection and protein extraction with RBS buffer with sonication were performed as described above. Extracts were quantified and equal concentrations were subjected for IP. Samples were pre-cleared using sepharose beads (Sigma) to reduce nonspecific binding. Co-IPs were performed as described for S2 cell experiments except α-FLAG M2 (Sigma), α-PER (GP5620), α-TIM (R3), and α-CLK (Santa Cruz Biotechnology H3107) antibodies were used. Samples were incubated with antibodies for 4 to 6 hours at 4°C on an end-over-end rotator. 20 μl of GammaBind Plus sepharose beads (GE) was added and incubation was continued for 2 hours. Samples were washed with RBS buffer three times, 10 minutes each, and immune complexes were resolved by SDS-PAGE as described above.

### Steady state mRNA expression analysis

Flies were entrained in 12 hr light:12 hr dark (LD) conditions at 25°C for three days and collected at four or six time-points on the fourth day. At least 40 flies of each genotype were collected per time point. Heads were collected using metal sieves. Total RNA extraction and quantitative PCR gene expression analysis were performed as previously described [86]. Gene-specific primers for *per*, *tim*, *vri*, and *brm* were designed to amplify fragments of around 150 bp near the 3’ end of the coding sequence for each target gene and optimized at an annealing temperature of 60°C. Primers for *clk* and *pdp1ε* were previously described [4]. Representative results are shown in Fig 3, S5 Fig, S6A Fig and S8 Fig. Additional biological replicates for expression analysis are presented in S12 Fig.

### Nascent pre-mRNA isolation

Nascent RNA was extracted as described [87–88] with modifications. Flies were entrained in LD conditions at 25°C for three days and collected at four time points on the fourth day. Fly heads were collected on dry ice. At least 300 μl of fly heads were used for nascent RNA extraction. Fly heads were homogenized into a fine powder using a liquid nitrogen chilled ceramic mortar and pestle, and mixed in 1.8 ml of homogenization buffer (10 mM Tris-HCl at pH 7.6, 10 mM KCl, 1.5 mM MgCl_2_, 0.8 M sucrose, 0.5 mM EDTA, 1 mM DTT, 1x protease inhibitor). Samples were dounced 15 times on ice with the loose pestle. The resulting lysate was then filtered through a 100 μm cell strainer in a 50 ml falcon tube and centrifuged at 300 g for 2 minutes. 700 μl of the supernatant was carefully removed and the remaining supernatant and pellet were resuspended and layered over 900 μl of sucrose cushion buffer (10 mM Tris-HCl at pH 7.5, 10 mM KCl, 1.5 mM MgCl_2,_ 1 M sucrose, 10% glycerol, 0.5 mM EDTA, 1 mM DTT, 1x protease inhibitor). Samples were spun at 11,000 rpm for 10 minutes. Pellets were resuspended in 1 ml of lysis buffer (20 mM Tris-HCl at pH 7.6, 150 mM NaCL, 2 mM EDTA, 1x protease inhibitor, 0.5 mM PMSF, 1 mM DTT, 0.5 U/ml RNAseOUT/SUPERase-In) and dounced 5 times with the tight pestle. After douncing, 1 ml of 2xNUN buffer (50 mM Tris-HCl at pH 7.6, 2M Urea, 2% NP-40, 600 mM NaCl, 2 mM DTT, 1x protease inhibitor, 0.5 mM PMSF, 0.5 U/ml SUPERase-In) was added drop-by-drop while gently vortexing. Samples were incubated on ice for 20 min, then centrifuged at 14,000 rpm for 30 min. Supernatant was removed and 500 μl of TRI Reagent (Sigma) was added to the pellet. Samples were incubated at 65°C for 15 min, then the DNA pellet was resuspended by gentle pipetting. Extraction using TRI Reagent, cDNA synthesis, and qPCR analysis (for comparison of control vs. flies expressing *brm* RNAi) was performed following previously described protocol [86].

### Droplet digital PCR

After nascent pre-mRNA isolation and cDNA synthesis, samples from *TUG(II)* and *BRM* ^*K804R*^ were diluted in nuclease-free water and ~10 ng of cDNA template was subjected to ddPCR. EvaGreen supermix reagent (Bio-Rad) was used following manufacturers protocol, and the QX200 Droplet Generator (Bio-Rad) was used to create 20,000 individual droplets in each reaction. Droplets were subjected to end-point PCR performed following manufacturers recommended cycling conditions. Primers for *per* and *tim* used for ddPCR were previously optimized for qPCR. Amplification of *cbp20* was used for normalization. Individual droplet fluorescence was measured on a QX200 ddPCR Droplet Reader (Bio-Rad) and analysis was performed using QuantaSoft software (Biorad). Technical triplicates from two biological replicates were performed. Data presented are unscaled expression levels normalized to *cbp20* expression. Error bars = SEM for biological replicates.

### Chromatin immunoprecipitation (ChIP)

Chromatin immunoprecipitation (ChIP) was performed based on published protocols [46, 51] with modifications. All buffers describe below, except CE buffer, contain protease inhibitors, 10 μg/μl aprotinin, 5 μg/μl leupeptin, 1 μg/μl pepstatin, and 0.5 mM PMSF. Flies entrained in 12 hr light:12 hr dark (LD) conditions at 25°C for three days were collected at four time-points (ZT) on the fourth day. 300 μl of fly heads were homogenized into a fine powder using a liquid nitrogen chilled ceramic mortar and pestle, mixed with 1.8 ml of NEB buffer (10 mM Tris-HCl at pH 8.0, 10 mM NaCl, 0.1 mM EGTA at pH 8.0, 0.5 mM EDTA at pH 8.0, .1 mM DTT, 0.5% Tergitol NP-10, 0.5 mM spermidine, 0.15 mM spermine, and 1x protease inhibitor (Sigma)), and homogenized with a dounce homogenizer (Kimble Chase) for 20 strokes using the loose “A” pestle. Homogenate was transferred to a 70 μm cell strainer placed in a 50 ml falcon tube and centrifuged at 300 g for 1 minute. The filtered homogenate was centrifuged at 6,700 rpm for 10 minutes to further remove cell debris. Pellets were resuspended in 1 ml of NEB and centrifuged at 11,500 rpm for 20 minutes on a sucrose gradient (0.6 ml of 1.6 M sucrose in NEB, 0.35 ml of 0.8 M sucrose in NEB). The nuclei-containing pellet was resuspended in 1 ml of NEB with 1% formaldehyde (diluted in *Drosophila* Schneider’s media (Life Technologies)) and cross-linked for 10 minutes at room temperature with rotation. Crosslinking was quenched by adding 150 μl of 1 M glycine and samples were rotated for 5 minutes at room temperature. Nuclei were collected by centrifugation at 6,700 rpm for 5 minutes, washed 2x with 1 ml NEB, and resuspended in 350 μl of sonication buffer (10 mM Tris-HCl at pH 7.5, 2 mM EDTA, 1% SDS, 0.2% Triton X-100, 0.5 mM spermidine, 0.15 mM spermine, and 1x protease inhibitor cocktail (Sigma)). Samples were sonicated 3x using a Diagenode Bioruptor on high setting for 5 minutes at 30 seconds on/off and then centrifuged at 10,000 rpm for 10 minutes. Supernatant was collected in two 130 μl aliquots for IP and 26 μl was collected for input and frozen at -80C for analysis. Sonicated chromatin was roughly 500 bp in length (<1000 bp). For each IP, 25 μl of a Protein G Dynabead slurry (Life Technologies) was washed twice in 75 μl of CW Buffer (50 mM Tris-HCl at pH 7.6, 1 mM EDTA, 1% Triton X-100, 0.1% DOC, 0.1% BSA, 0.5 M KCl in PBS, 150 mM NaCl, 0.5 M EGTA, 0.1% SDS, and 1x protease inhibitor (Sigma)). Beads were captured using a magnetic stand (Millipore) to allow for buffer removal. After the last wash, 75 μl of CW buffer was added to the beads along with the appropriate antibody and incubated with rotation for 2 hours at 4°C. Amount of antibodies used for ChIP is as follows: α-PER (GP5620) (20 μg/ml); α-CLK (15 μg/ml); α-H3 (10 μg/ml); α-RNAPII (10 μg/ml), α-FLAG (10 μg/ml). Following incubation, beads were collected and resuspended in 22 μl of CW buffer. 20 μl of this slurry was added to sonicated chromatin IP aliquots that were diluted 10-fold with IP buffer (50 mM Tris-HCl at pH 7.6, 2 mM EDTA, 1% Triton X-100, 0.1% DOC, 150 mM NaCl, 0.5 mM EGTA, and 1x protease inhibitor) and incubated for 2 hours at 4°C. Beads were captured and washed for 30 minutes 2x in 1 ml of CW buffer, once in LW buffer (10 mM Tris-HCl at pH 8.0, 0.25 M LiCl, 0.5% NP40, 0.5% DOC, 1mM EDTA), and once in TE buffer for 4 minutes. Contents were then transferred to new LoBind tubes. Supernatant was removed and 150 μl of CE buffer (50 mM Tris-HCl at pH 8.0, 10 mM EDTA, 1% SDS, 1 mM DTT, 0.1 mg/ml proteinase K, 50 mM NaCl, and 0.05 mg/ml RNase A) was added. CE buffer (150 μl) was also added to input samples. All samples were incubated for 2 hours at 37°C. Beads were then removed from IP samples and supernatant was de-crosslinked overnight at 65°C. DNA was eluted using the Qiagen PCR purification kit and subjected to qPCR. At least three technical replicates of qPCR were performed for each biological ChIP replicate and three biological replicates were performed for CLK, PER, H3, and RNAPII assays. Background binding to a non-specific antibody (α-V5; Life Technologies) at 10μg/ml bound to Dynabeads was subtracted from input samples and results are presented as the percentage of the input samples. For each assay, at least three biological replicates were performed, with technical triplicates for the qPCR step for each biological replicate. The technical qPCR triplicates were averaged for each biological replicate as no significant differences were found between the technical replicates, and the error bars represent SEM calculated from variance between biological replicates. Two-tailed t-tests were used to determine statistical differences (P < 0.05) between control and experimental treatment at each ZT.

### Splicing assay

To detect splicing efficiency in *period*, cDNA was generated using methods described in [86] and used as DNA templates for semi-quantitative PCR as described in [67]. The PCR program was set to run for 23 cycles to ensure that amplicons were still in the log-linear phase of amplification. Primers designed to flank the 8^th^ intron in the 3’UTR of *per* were used to assay splicing efficiency between WT and *brm* RNA*i* mutants. For normalization, we also included primers that amplify the non-cycling *cbp20* gene. PCR products were separated and visualized by gel electrophoresis on 2% agarose gels by staining with Gelstar (Cambrex Co.) and DNA bands were quantified using a ChemiDoc MP with Image Lab software (Bio-Rad).

## Supporting Information

S1 Fig Co-immunoprecipitation (Co-IP) using proteins extracted from whole heads of flies expressing *BRM* ^*RFL*^ in *tim*-expressing cells (*TUG(II)* driver) confirms interactions of BRM and clock proteins observed in S2 cells. *(A)* Protein extracts were immunoprecipitated using α-FLAG to pull down FLAG-BRM. Immunocomplexes were subjected to western blotting to detect protein interactions between BRM and CLK as well as BRM and TIM at the indicated time points (ZT) over a circadian cycle (LD). Antibodies used were α-FLAG to detect BRM (top), α-CLK (middle), and α-TIM (bottom). *(B)* Western blotting of lysate representing inputs for co-IP. Bracket denotes all isoforms of CLK with different electrophoretic mobility. All flies were entrained in 12 hr light:12 hr dark (LD) conditions and samples were collected on LD4 at the indicated time points (ZT). Data shown are representative of two biological replicates. Quantification of signal intensity representing interaction of BRM to *(C)* CLK and *(D)* TIM. Quantification was performed with NIH ImageJ software. The signal from each co-IP reaction is normalized to corresponding signal intensity of input. Shown are the scaled average values from two biological replicates. All values were scaled where the highest normalized signal equals to 1. Error bars = SEM. *(E)* Western blot detecting CLK from α-FLAG pull-down of BRM (left, top, pink signal) overlaid on western blot of CLK lysates (left, bottom, grey scale) to illustrate preferential binding of BRM to hypophosphorylated CLK.
(TIF)

S2 Fig Loss of rhythmicity in flies expressing *brm* ^*K804R*^ transgene is gradual upon transition into constant DD condition. Eduction graphs showing the average activity in day two through day four of LD (LD2-4) entrainment followed by daily activity during DD1, DD2, and DD3 in *(A) w* ^*1118*^ control, *(B) w*; *tim-UAS-GAL4* (*TUG(II*)) driver line, *(C) per* ^*0*^, *(D)* progenies of *w* ^*1118*^ crossed to *TUG(II*), *(E) UAS*-*brm* ^*K804R*^ responder line, and *(F)* progenies of *UAS*-*brm* ^*K804R*^ crossed to *TUG(II)*. Arrows indicate the presence of weak activity peaks in DD1 and DD2 that are undetected in arrhythmic *per* ^*0*^ mutants.
(TIF)

S3 Fig ChIP with α-FLAG antibody on *TUG(II)* flies that are not expressing FLAG epitope tag shows specificity of FLAG antibody to FLAG-BRM protein. *TUG(II)* and *BRM* ^*RFL*^ flies were collected at ZT4 and ZT16 and processed for ChIP-qPCR using primers to amplify the *per* promoter. Data shown are from two biological replicates, with technical triplicates performed during qPCR for each biological replicate. Error bars = SEM of biological replicates.
(TIF)

S4 Fig BRM shows preferential binding to the *per* promoter over a gene body region 5.2kb downstream of the TSS. ChIP assays using α-FLAG to detect FLAG-BRM binding to *per* at the CRS region compared to FLAG-BRM binding to a region on the *per* gene body at ZT4 and ZT16. All flies were entrained in 12 hr light:12 hr dark (LD) conditions and samples were collected on LD4 at the indicated time points (ZT). Data presented are representative of three biological replicates. Error bars = SEM of technical triplicates for one biological replicate.
(TIF)

S5 Fig BRM regulates CLK-dependent transcription in *Drosophila*. *(A) pdp1ε and vrille* expression in *TUG* control and flies expressing *brm* RNA*i*. *(B) pdp1ε and vrille* expression in *TUG(II)* and flies expressing *brm* ^*K804R*^. Gene expression analysis was performed with quantitative real-time PCR using SYBR green chemistry. Steady state mRNA levels at six time points for *(A)* and four time points for *(B)* over a circadian cycle were normalized to non-cycling *cbp20* levels, and expressed as a fraction of the peak expression level (peak = 1). All flies were entrained in 12 hr light:12 hr dark (LD) conditions and samples were collected on LD4 at the indicated time points (ZT). Experiments were performed three times (except two biological replicates were performed to assay *vri* expression in flies expressing *brm* ^*K804R*^). Error bars = SEM for technical triplicates of representative biological replicate.
(TIF)

S6 Fig BRM regulates nascent pre-mRNA expression levels of *per* and *tim*. *(A) per and tim* expression in *TUG* control and flies expressing *brm* RNA*i*. Gene expression was analyzed using qPCR with SYBR green chemistry. Nascent mRNA levels were normalized to non-cycling *cbp20*, and expressed as a fraction of the peak expression level (peak = 1). Data shown is representative of two biological replicates (technical triplicates were performed at the qPCR step for each biological replicate). Error bars = SEM of technical triplicates for representative biological replicate. *(B) per and tim* expression in *TUG(II)* and flies expressing *brm* ^*K804R*^. Gene expression analysis was performed using droplet digital PCR (ddPCR; Biorad QX200) to achieve absolute quantification. Shown are gene expression levels normalized to non-cycling *cbp20* levels, and expressed as a fraction of the peak expression level (peak = 1). Experiments were performed for two biological replicates. Error bars = SEM for biological replicates. Significant differences are observed for ZT4, 10, and 22 for *per*, and ZT4, 16, and 22 for *tim* (p<0.05). All flies were entrained in 12 hr light:12 hr dark (LD) conditions and samples were collected on LD4 at the indicated time points (ZT).
(TIF)

S7 Fig CLK and PER ChIP assays show binding specificity to target genes. *(A)* ChIP detecting CLK binding to a CLK target (*per* promoter) as compared to a non-CLK target (*clkC2C3* promoter region). *(B)* ChIP detecting PER binding to a PER target (*per* promoter) as compared to a non-PER target (*clkC2C3* promoter region). Control *TUG(II)* flies were entrained in 12 hr light:12 hr dark (LD) conditions and samples were collected on LD4 at the indicated time points (ZT). Data presented are from two biological replicates, with technical triplicates performed during qPCR for each biological replicate. Error bars = SEM of biological replicates.
(TIF)

S8 Fig The *brahma* complex regulates *Clk* expression. Gene expression analysis *of Clk* in flies expressing *brm* RNA*i* (top panel) and *brm* ^*K804R*^ (bottom panel) in *tim*-expressing cells as compared to the respective *TUG* and *TUG(II)* controls. Gene expression analysis was performed with quantitative real-time PCR using SYBR green chemistry. Steady state *Clk* mRNA levels were normalized to non-cycling *cbp20* levels, and expressed as a fraction of the peak expression level (peak = 1). All flies were entrained in 12 hr light:12 hr dark (LD) conditions and samples were collected on LD4 at the indicated time points (ZT). Experiments were performed three times and representative results are shown. Error bars = SEM for technical triplicates for representative biological replicate.
(TIF)

S9 Fig PER and CLK protein expression in *brm* mutants. *(A)* PER and CLK expression in flies expressing *brm* ^*K804R*^ in *tim*-expressing cells as compared to *TUG(II)* control. Whereas PER levels show a slight decrease in *brm* ^*K804R*^ expressing flies, especially at ZT16, CLK levels show a much more apparent reduction at all time points except ZT4. Data presented are representative of at least three biological experiments. *(B)* Quantification of CLK levels in *TUG(II)* and *brm* ^*K804R*^. Quantification was performed using Image Lab software (Bio-Rad) and normalized to HSP70. Error bars = SEM for three biological replicates. *(C)* PER and CLK expression in flies expressing *brm* RNA*i* in *tim*-expressing cells compared to *TUG* control. Both PER and CLK show increased expression in flies expressing *brm* RNAi. *(D)* Quantification of CLK levels in *TUG* and *brm* RNA*i*. Quantification was performed using Image Lab software and normalized to nonspecific band present on the α-CLK Western blot. Error bars = SEM for three biological replicates. All flies were entrained in 12 hrs:12 hrs light:dark cycle and samples were collected on LD4 at the indicated time points (ZT).
(TIF)

S10 Fig Primer location and sequences. *(A)* Schematic of primer locations used for ChIP analysis on *per* and *tim* loci. Regions are numbered relative to the TSS (+1). *(B)* Full list of primer sequences used for ChIP assays. *(C)* Primer sequences used for gene expression analysis. All primers were optimized to anneal at 60°C for quantitative real-time PCR analysis.
(TIF)

S11 Fig Alternative splicing of *per* transcripts is not affected upon *brm* RNA*i* knock down. Agarose gel electrophoresis of semi-quantitative RT-PCR products to measure the relative levels of spliced *per* (middle band) relative to unspliced *per* (top band) in *(A)* control *TUG* driver line and *(B)* flies expressing *brm* RNA*i* in *tim-*expressing cells *(TUG)*. Non-cycling *cbp20* transcript (bottom band) was used for control and normalization. (C) Quantification of unspliced *per* transcripts out of total (spliced and unspliced) in control *TUG* flies. (D) Quantification of unspliced *per* transcripts out of total in *brm* RNA*i* flies. Flies were entrained for three full days in 12 hr light:12 hr dark (LD) conditions at 25°C and collected on the fourth day at the indicated time points (ZT) and frozen immediately on dry ice.
(TIF)

S12 Fig Additional biological replicates of gene expression analysis confirm that BRM regulates CLK-dependent transcription in *Drosophila*. (A) Steady state mRNA expression analysis *of per*, *tim*, *vri*, *and pdp1ε* in flies expressing *brm* RNA*i* or *brm* ^*K804R*^ in *tim*-expressing cells as compared to the respective *TUG* and *TUG(II)* controls. (B) Nascent pre-mRNA expression analysis of *per* and *tim* in flies expressing *brm* RNA*i* in *tim*-expressing cells as compared to *TUG* control. Gene expression analysis was performed with quantitative real-time PCR using SYBR green chemistry. These experiments represent additional biological replicates in support of the results shown in Fig 3, S5 Fig, and S6A Fig.
(TIF)
