# Supplementary figures and images for: The Catalytic and Non-catalytic Functions of the Brahma Chromatin-Remodeling Protein Collaborate to Fine-Tune Circadian Transcription in Drosophila
Source: PLoS Genet. 2015 Jul 1;11(7):e1005307. doi: 10.1371/journal.pgen.1005307 (PMC4488936; doi:10.1371/journal.pgen.1005307)

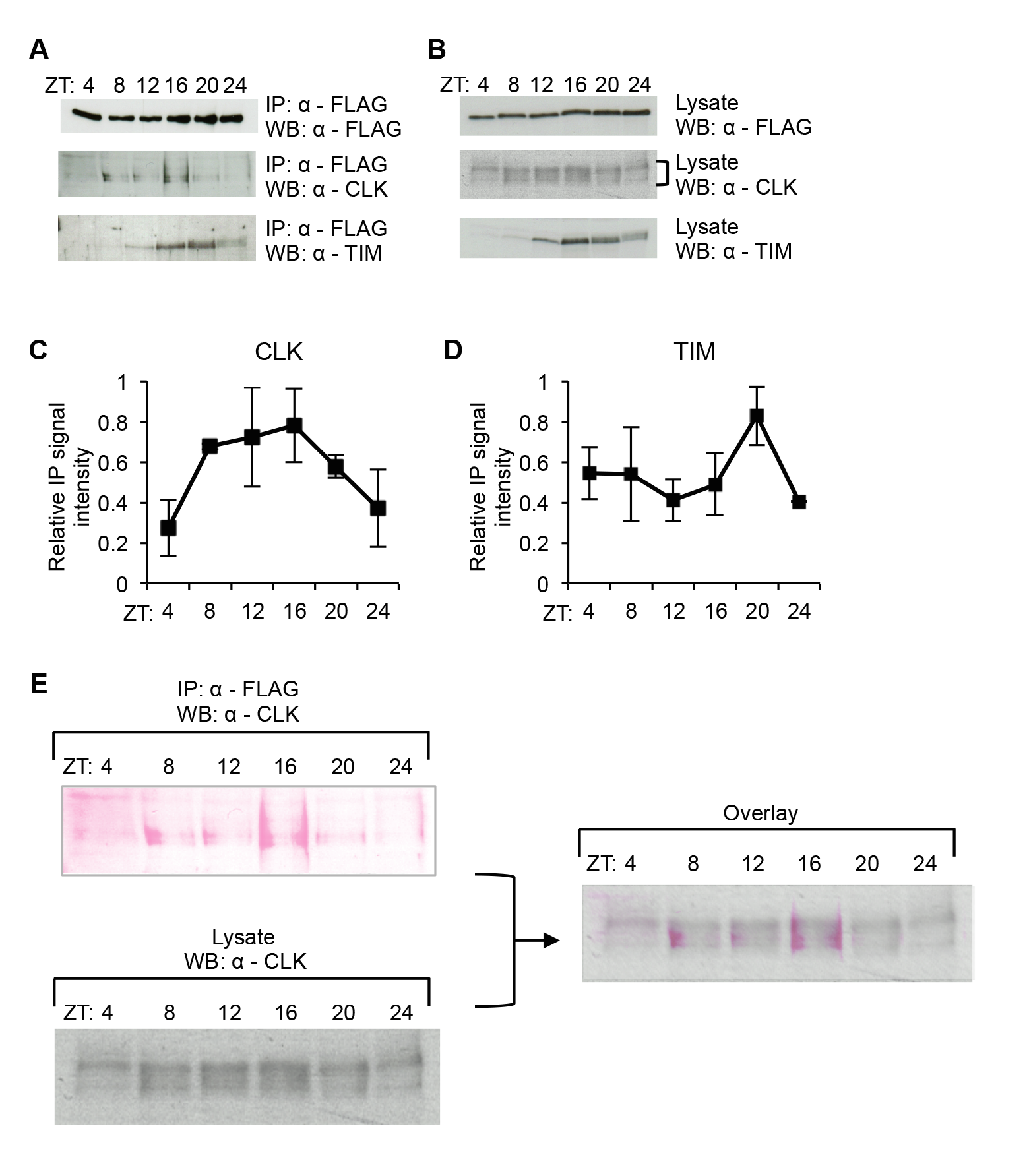

Supplement: S1 Fig — (A) Protein extracts were immunoprecipitated using α-FLAG to pull down FLAG-BRM. Immunocomplexes were subjected to western blotting to detect protein interactions between BRM and CLK as well as BRM and TIM at the indicated time points (ZT) over a circadian cycle (LD). Antibodies used were α-FLAG to detect BRM (top), α-CLK (middle), and α-TIM (bottom). (B) Western blotting of lysate representing inputs for co-IP. Bracket denotes all isoforms of CLK with different electrophoretic mobility. All flies were entrained in 12 hr light:12 hr dark (LD) conditions and samples were collected on LD4 at the indicated time points (ZT). Data shown are representative of two biological replicates. Quantification of signal intensity representing interaction of BRM to (C) CLK and (D) TIM. Quantification was performed with NIH ImageJ software. The signal from each co-IP reaction is normalized to corresponding signal intensity of input. Shown are the scaled average values from two biological replicates. All values were scaled where the highest normalized signal equals to 1. Error bars = SEM. (E) Western blot detecting CLK from α-FLAG pull-down of BRM (left, top, pink signal) overlaid on western blot of CLK lysates (left, bottom, grey scale) to illustrate preferential binding of BRM to hypophosphorylated CLK. (TIF) [file pgen.1005307.s001.tif]

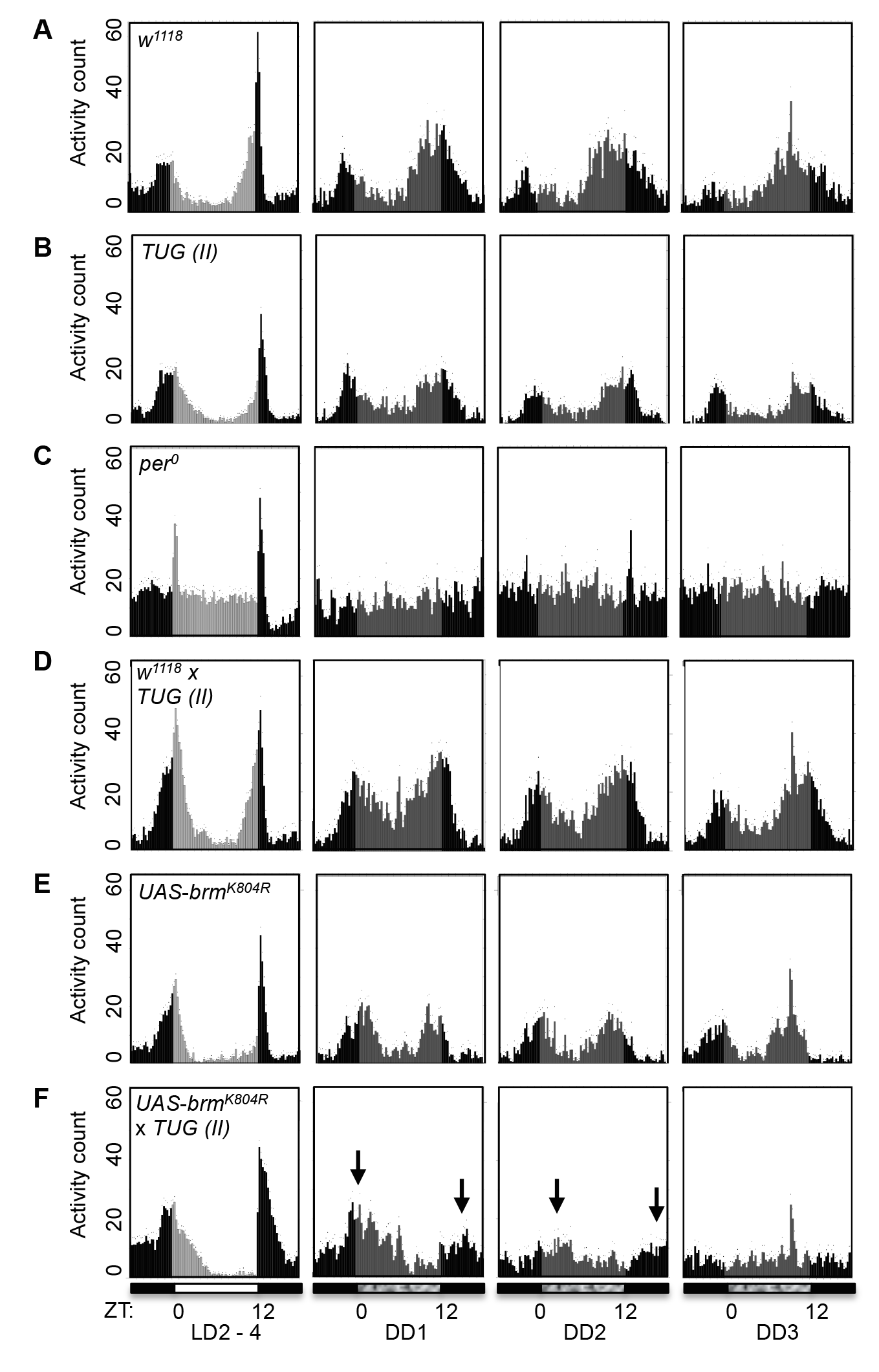

Supplement: S2 Fig — Eduction graphs showing the average activity in day two through day four of LD (LD2-4) entrainment followed by daily activity during DD1, DD2, and DD3 in (A) w 1118 control, (B) w; tim-UAS-GAL4 (TUG(II)) driver line, (C) per 0, (D) progenies of w 1118 crossed to TUG(II), (E) UAS-brm K804R responder line, and (F) progenies of UAS-brm K804R crossed to TUG(II). Arrows indicate the presence of weak activity peaks in DD1 and DD2 that are undetected in arrhythmic per 0 mutants. (TIF) [file pgen.1005307.s002.tif]

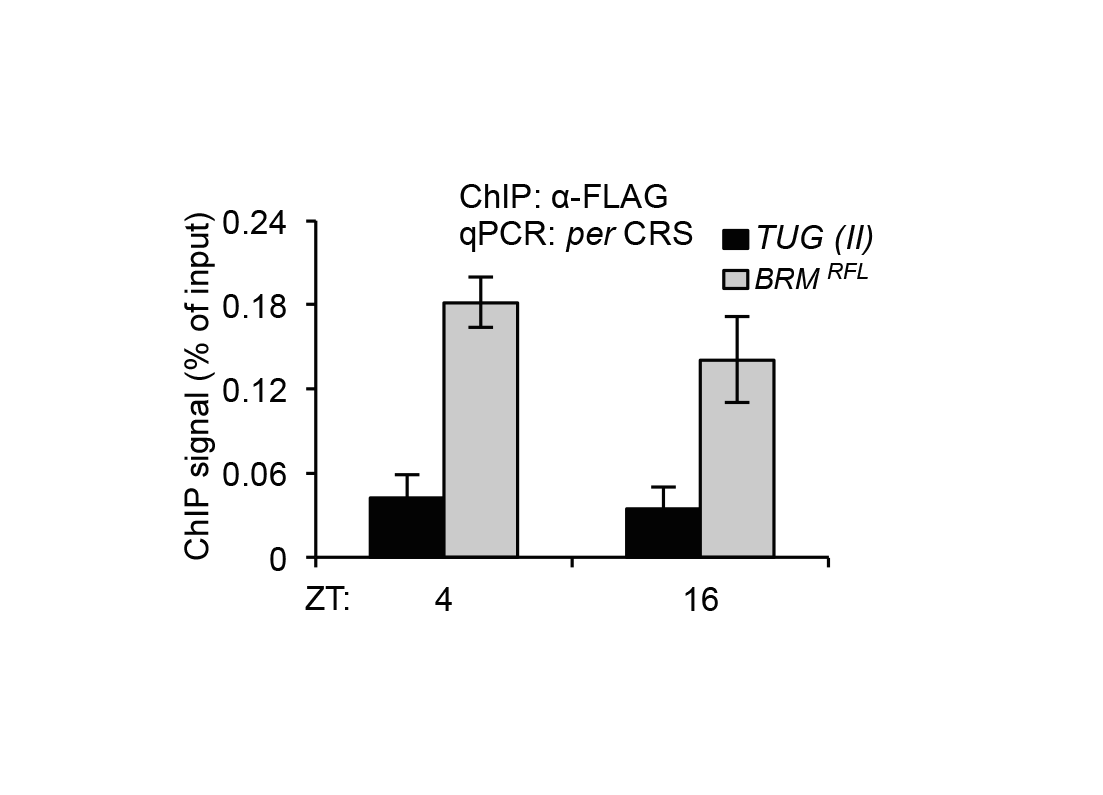

Supplement: S3 Fig — TUG(II) and BRM RFL flies were collected at ZT4 and ZT16 and processed for ChIP-qPCR using primers to amplify the per promoter. Data shown are from two biological replicates, with technical triplicates performed during qPCR for each biological replicate. Error bars = SEM of biological replicates. (TIF) [file pgen.1005307.s003.tif]

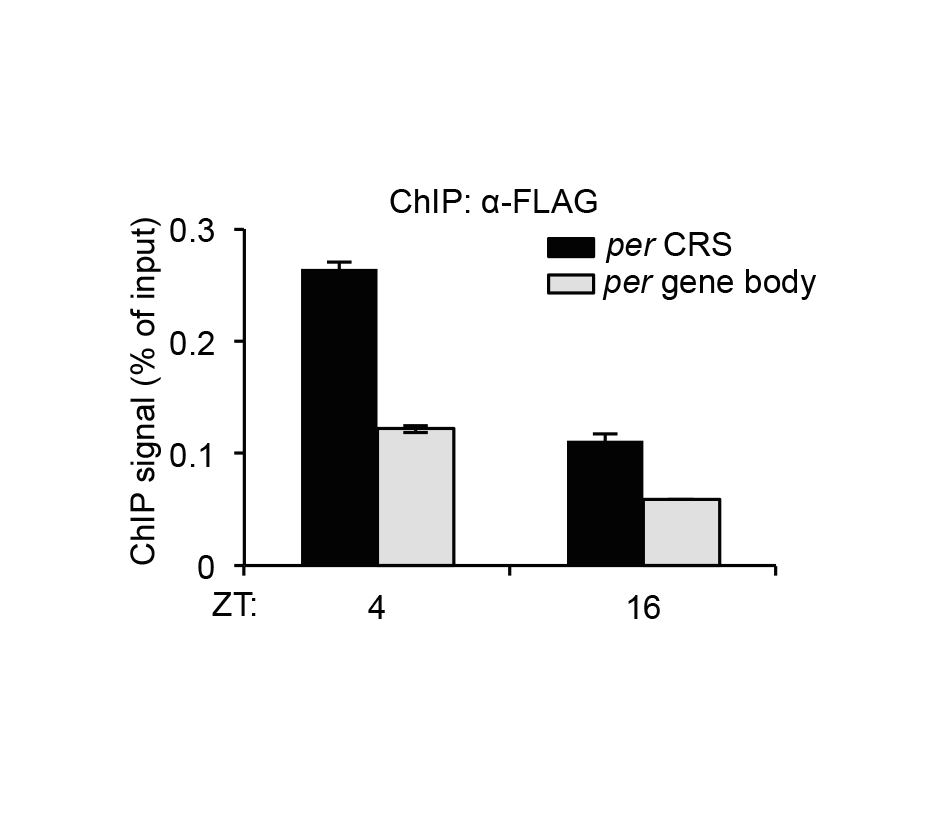

Supplement: S4 Fig — ChIP assays using α-FLAG to detect FLAG-BRM binding to per at the CRS region compared to FLAG-BRM binding to a region on the per gene body at ZT4 and ZT16. All flies were entrained in 12 hr light:12 hr dark (LD) conditions and samples were collected on LD4 at the indicated time points (ZT). Data presented are representative of three biological replicates. Error bars = SEM of technical triplicates for one biological replicate. (TIF) [file pgen.1005307.s004.tif]

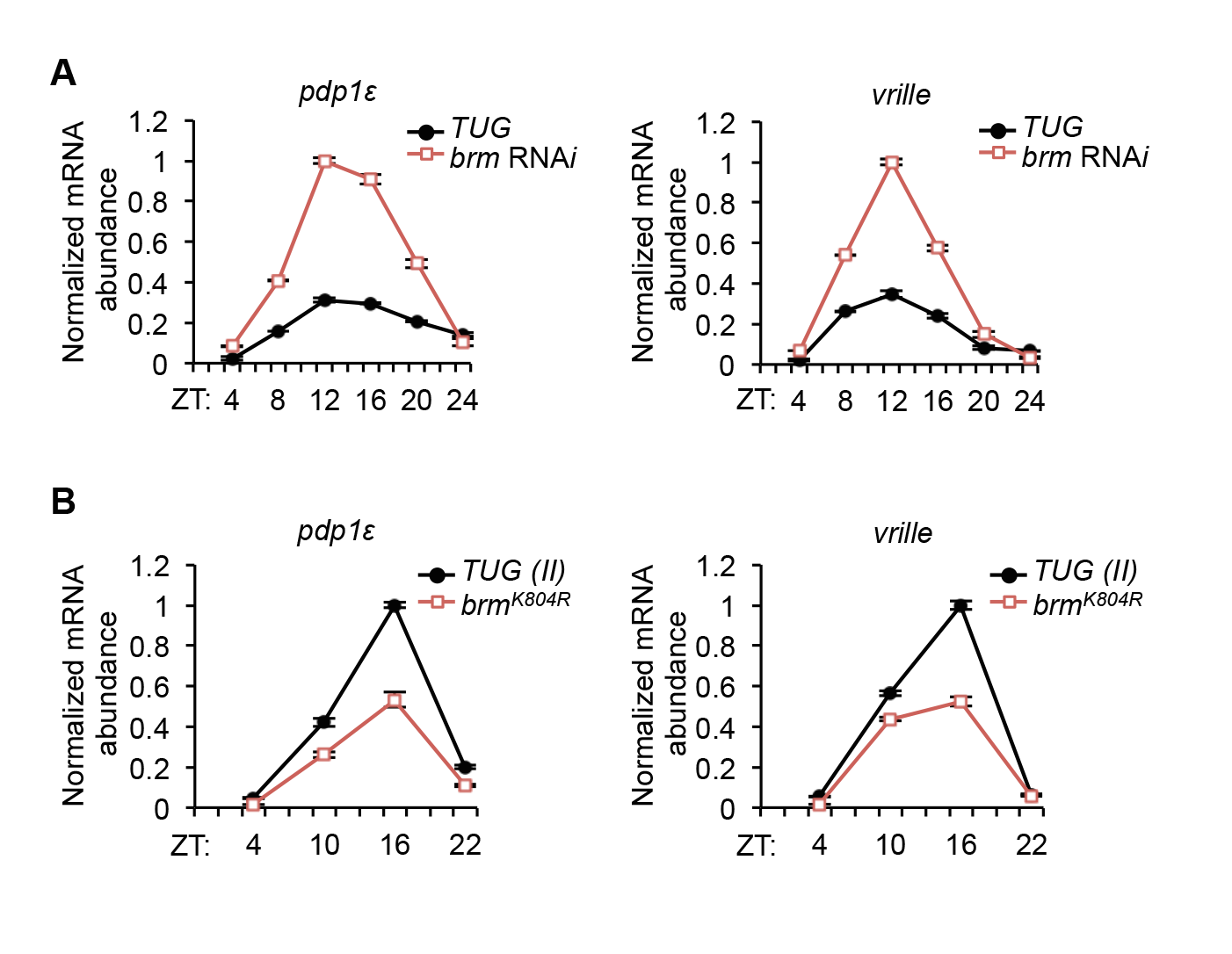

Supplement: S5 Fig — (A) pdp1ε and vrille expression in TUG control and flies expressing brm RNAi. (B) pdp1ε and vrille expression in TUG(II) and flies expressing brm K804R. Gene expression analysis was performed with quantitative real-time PCR using SYBR green chemistry. Steady state mRNA levels at six time points for (A) and four time points for (B) over a circadian cycle were normalized to non-cycling cbp20 levels, and expressed as a fraction of the peak expression level (peak = 1). All flies were entrained in 12 hr light:12 hr dark (LD) conditions and samples were collected on LD4 at the indicated time points (ZT). Experiments were performed three times (except two biological replicates were performed to assay vri expression in flies expressing brm K804R). Error bars = SEM for technical triplicates of representative biological replicate. (TIF) [file pgen.1005307.s005.tif]

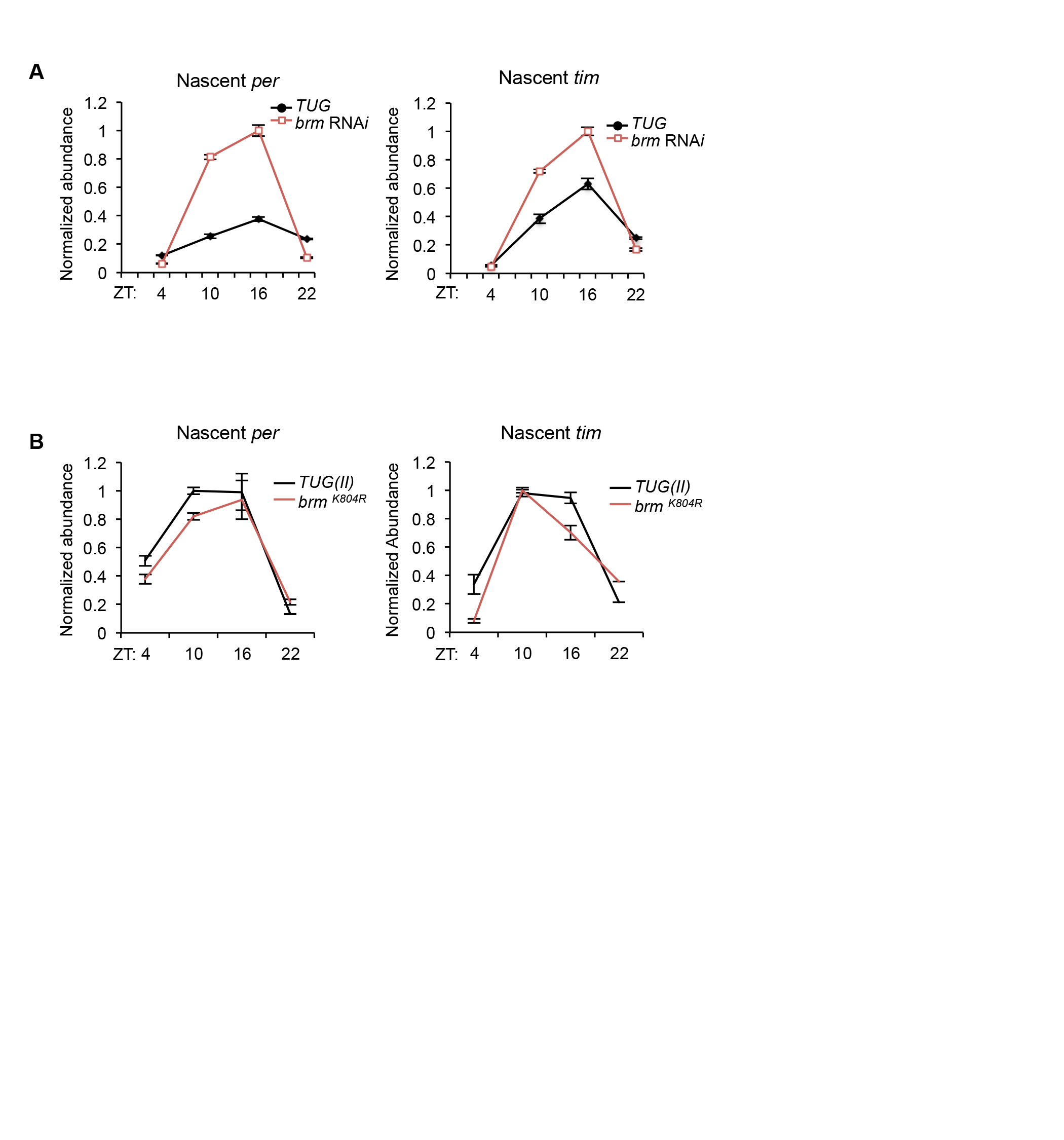

Supplement: S6 Fig — (A) per and tim expression in TUG control and flies expressing brm RNAi. Gene expression was analyzed using qPCR with SYBR green chemistry. Nascent mRNA levels were normalized to non-cycling cbp20, and expressed as a fraction of the peak expression level (peak = 1). Data shown is representative of two biological replicates (technical triplicates were performed at the qPCR step for each biological replicate). Error bars = SEM of technical triplicates for representative biological replicate. (B) per and tim expression in TUG(II) and flies expressing brm K804R. Gene expression analysis was performed using droplet digital PCR (ddPCR; Biorad QX200) to achieve absolute quantification. Shown are gene expression levels normalized to non-cycling cbp20 levels, and expressed as a fraction of the peak expression level (peak = 1). Experiments were performed for two biological replicates. Error bars = SEM for biological replicates. Significant differences are observed for ZT4, 10, and 22 for per, and ZT4, 16, and 22 for tim (p<0.05). All flies were entrained in 12 hr light:12 hr dark (LD) conditions and samples were collected on LD4 at the indicated time points (ZT). (TIF) [file pgen.1005307.s006.tif]

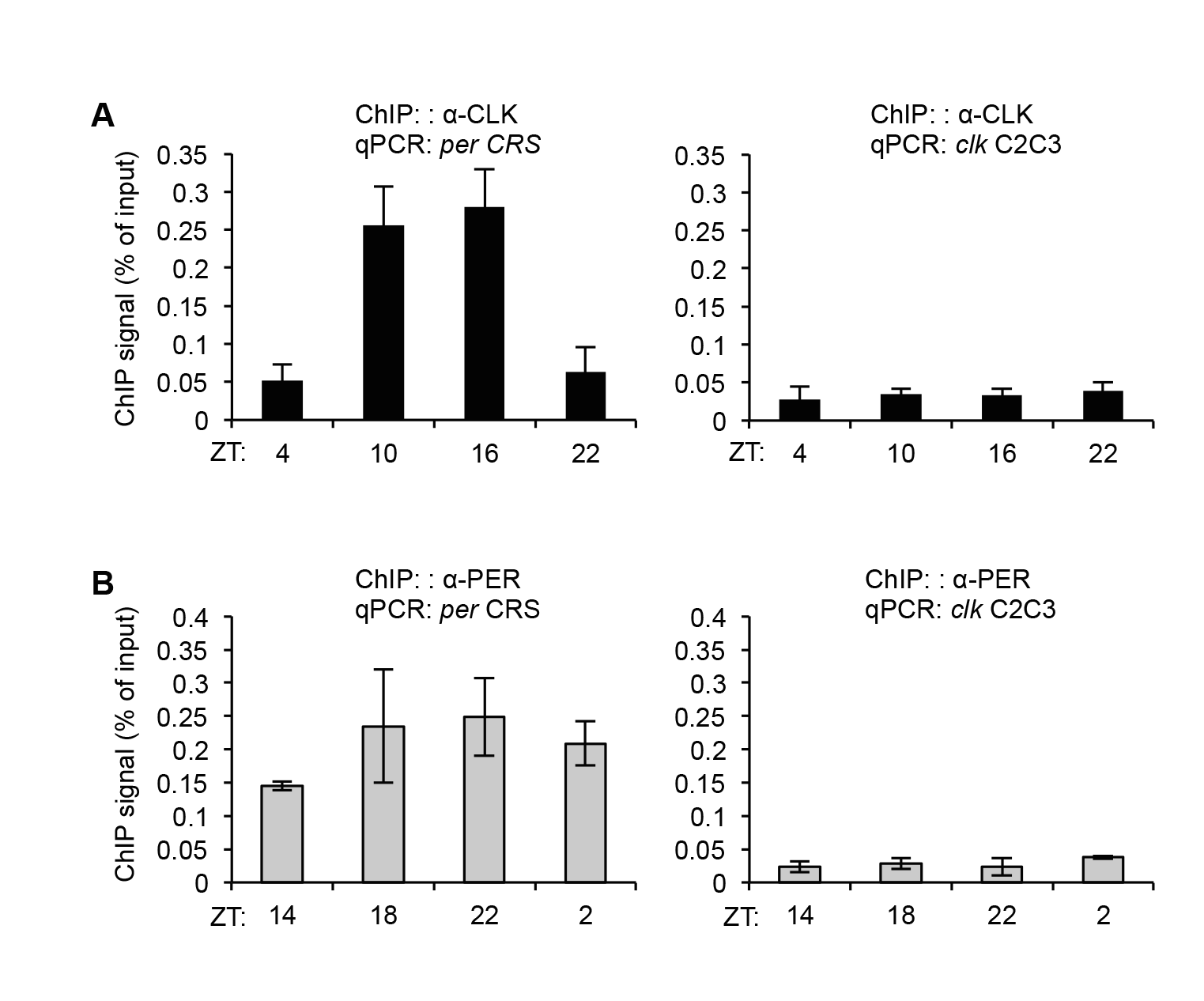

Supplement: S7 Fig — (A) ChIP detecting CLK binding to a CLK target (per promoter) as compared to a non-CLK target (clkC2C3 promoter region). (B) ChIP detecting PER binding to a PER target (per promoter) as compared to a non-PER target (clkC2C3 promoter region). Control TUG(II) flies were entrained in 12 hr light:12 hr dark (LD) conditions and samples were collected on LD4 at the indicated time points (ZT). Data presented are from two biological replicates, with technical triplicates performed during qPCR for each biological replicate. Error bars = SEM of biological replicates. (TIF) [file pgen.1005307.s007.tif]

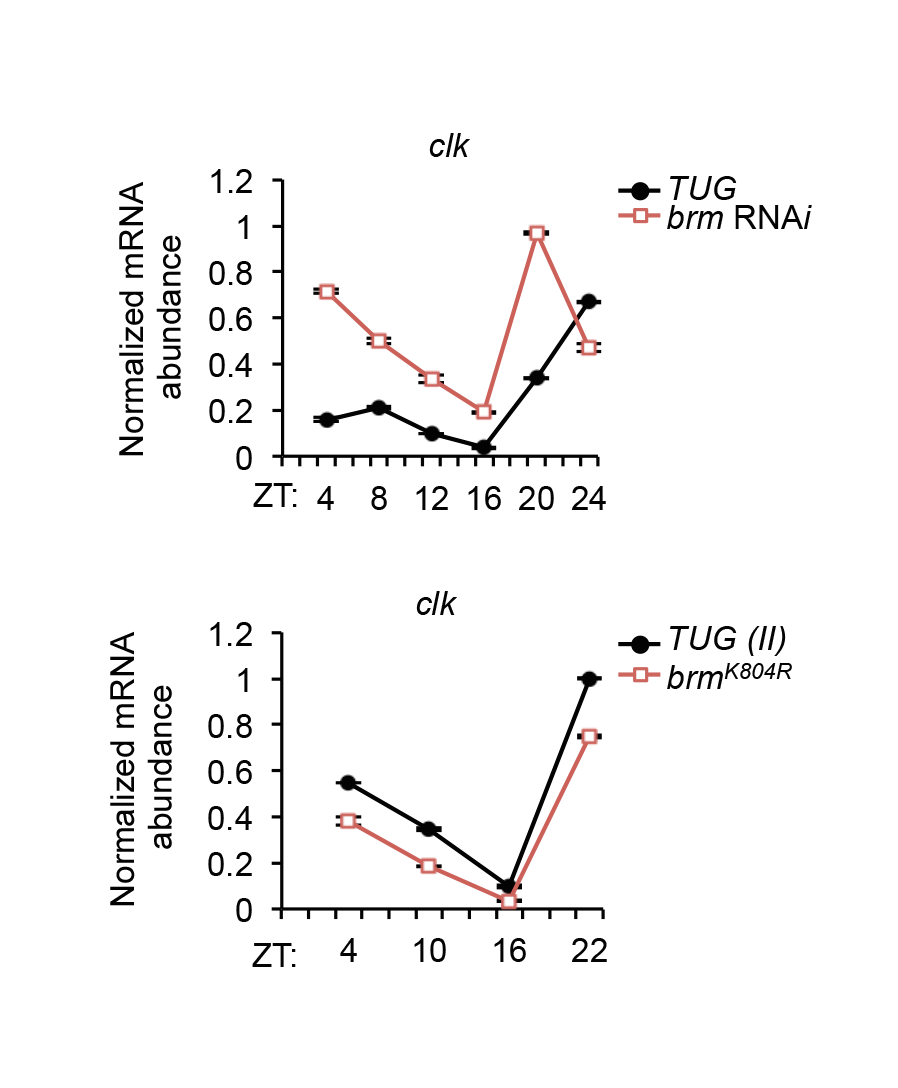

Supplement: S8 Fig — Gene expression analysis of Clk in flies expressing brm RNAi (top panel) and brm K804R (bottom panel) in tim-expressing cells as compared to the respective TUG and TUG(II) controls. Gene expression analysis was performed with quantitative real-time PCR using SYBR green chemistry. Steady state Clk mRNA levels were normalized to non-cycling cbp20 levels, and expressed as a fraction of the peak expression level (peak = 1). All flies were entrained in 12 hr light:12 hr dark (LD) conditions and samples were collected on LD4 at the indicated time points (ZT). Experiments were performed three times and representative results are shown. Error bars = SEM for technical triplicates for representative biological replicate. (TIF) [file pgen.1005307.s008.tif]

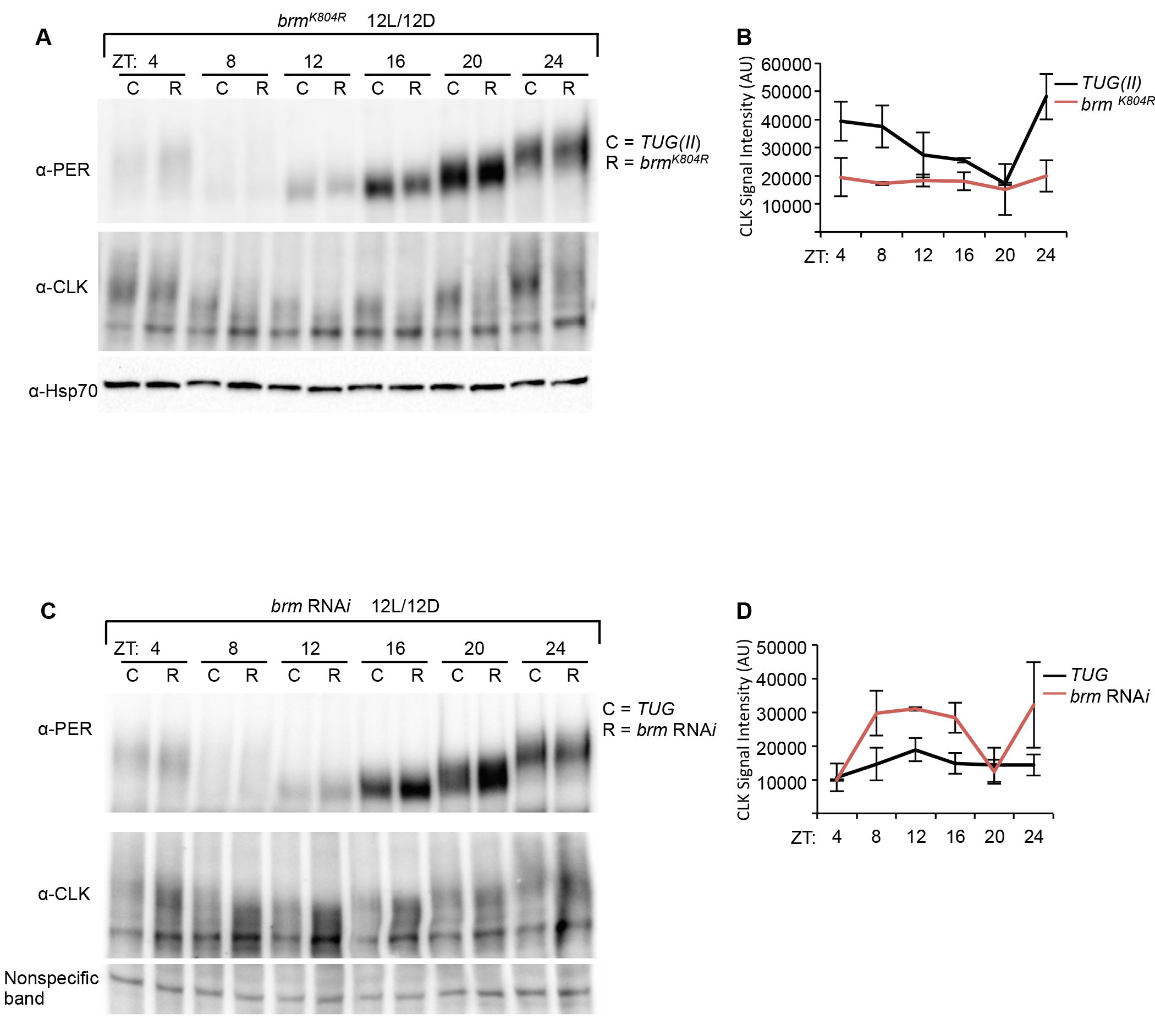

Supplement: S9 Fig — (A) PER and CLK expression in flies expressing brm K804R in tim-expressing cells as compared to TUG(II) control. Whereas PER levels show a slight decrease in brm K804R expressing flies, especially at ZT16, CLK levels show a much more apparent reduction at all time points except ZT4. Data presented are representative of at least three biological experiments. (B) Quantification of CLK levels in TUG(II) and brm K804R. Quantification was performed using Image Lab software (Bio-Rad) and normalized to HSP70. Error bars = SEM for three biological replicates. (C) PER and CLK expression in flies expressing brm RNAi in tim-expressing cells compared to TUG control. Both PER and CLK show increased expression in flies expressing brm RNAi. (D) Quantification of CLK levels in TUG and brm RNAi. Quantification was performed using Image Lab software and normalized to nonspecific band present on the α-CLK Western blot. Error bars = SEM for three biological replicates. All flies were entrained in 12 hrs:12 hrs light:dark cycle and samples were collected on LD4 at the indicated time points (ZT). (TIF) [file pgen.1005307.s009.tif]

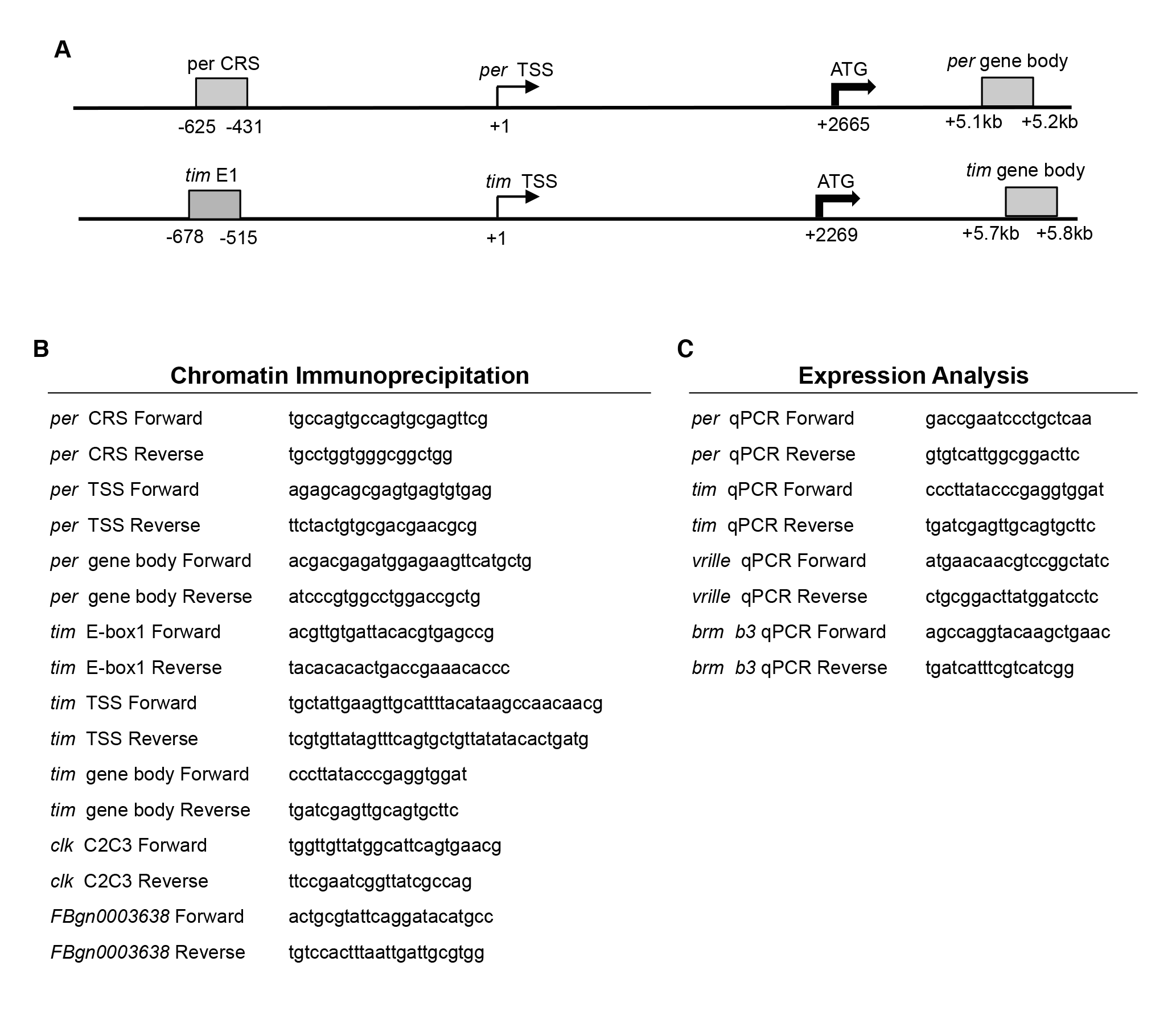

Supplement: S10 Fig — (A) Schematic of primer locations used for ChIP analysis on per and tim loci. Regions are numbered relative to the TSS (+1). (B) Full list of primer sequences used for ChIP assays. (C) Primer sequences used for gene expression analysis. All primers were optimized to anneal at 60°C for quantitative real-time PCR analysis. (TIF) [file pgen.1005307.s010.tif]

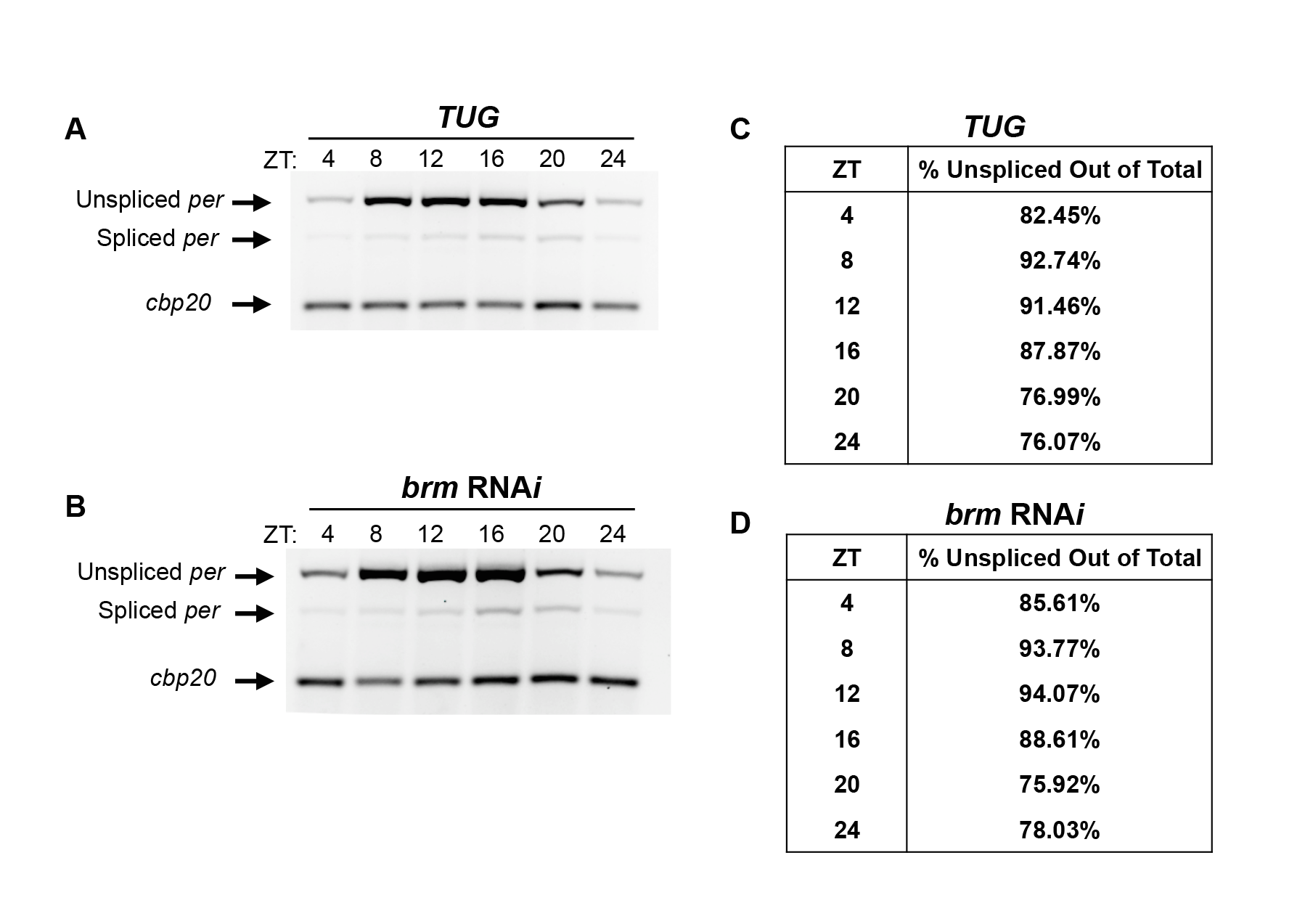

Supplement: S11 Fig — Agarose gel electrophoresis of semi-quantitative RT-PCR products to measure the relative levels of spliced per (middle band) relative to unspliced per (top band) in (A) control TUG driver line and (B) flies expressing brm RNAi in tim-expressing cells (TUG). Non-cycling cbp20 transcript (bottom band) was used for control and normalization. (C) Quantification of unspliced per transcripts out of total (spliced and unspliced) in control TUG flies. (D) Quantification of unspliced per transcripts out of total in brm RNAi flies. Flies were entrained for three full days in 12 hr light:12 hr dark (LD) conditions at 25°C and collected on the fourth day at the indicated time points (ZT) and frozen immediately on dry ice. (TIF) [file pgen.1005307.s011.tif]

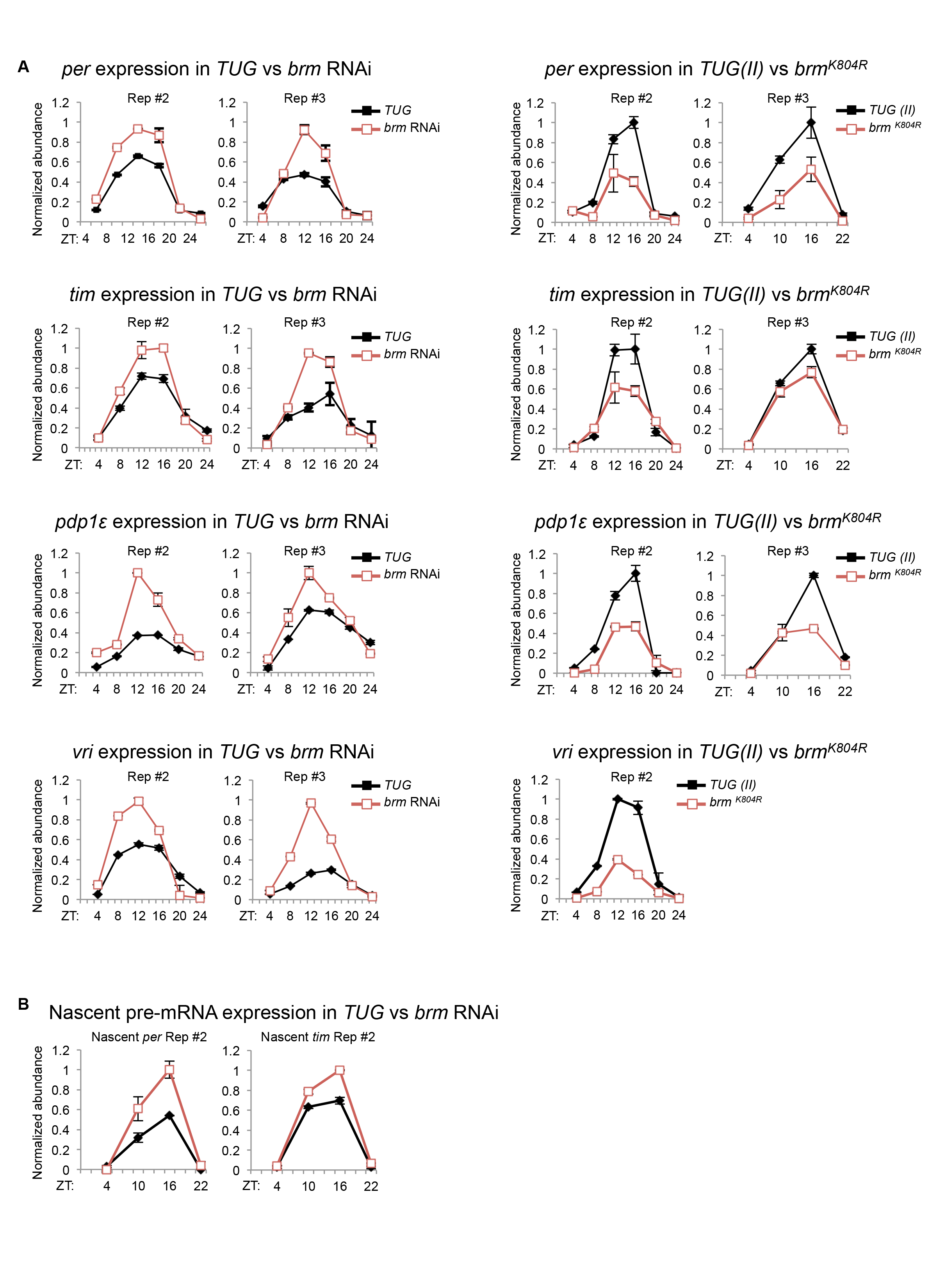

Supplement: S12 Fig — (A) Steady state mRNA expression analysis of per, tim, vri, and pdp1ε in flies expressing brm RNAi or brm K804R in tim-expressing cells as compared to the respective TUG and TUG(II) controls. (B) Nascent pre-mRNA expression analysis of per and tim in flies expressing brm RNAi in tim-expressing cells as compared to TUG control. Gene expression analysis was performed with quantitative real-time PCR using SYBR green chemistry. These experiments represent additional biological replicates in support of the results shown in Fig 3, S5 Fig, and S6A Fig. (TIF) [file pgen.1005307.s012.tif]
